# Annexin A6 and NPC1 regulate LDL-inducible cell migration and distribution of focal adhesions

**DOI:** 10.1038/s41598-021-04584-y

**Published:** 2022-01-12

**Authors:** Jaimy Jose, Monira Hoque, Johanna Engel, Syed S. Beevi, Mohamed Wahba, Mariya Ilieva Georgieva, Kendelle J. Murphy, William E. Hughes, Blake J. Cochran, Albert Lu, Francesc Tebar, Andrew J. Hoy, Paul Timpson, Kerry-Anne Rye, Carlos Enrich, Carles Rentero, Thomas Grewal

**Affiliations:** 1 School of Pharmacy, Faculty of Medicine and Health, University of Sydney, Sydney, NSW 2006 Australia; 2 Cancer Research Program, Garvan Institute of Medical Research and Kinghorn Cancer Centre, St. Vincent’s Clinical School, Faculty of Medicine, University of New South Wales, Sydney, NSW 2010 Australia; 3 Children’s Medical Research Institute, University of Sydney, Westmead, NSW 2145 Australia; 4 School of Medical Sciences, University of New South Wales, Sydney, NSW 2052 Australia; 5 Departament de Biomedicina, Unitat de Biologia Cellular, Facultat de Medicina i Ciències de la Salut, Universitat de Barcelona, 08036 Barcelona, Spain; 6 Centre de Recerca Biomèdica CELLEX, Institut d’Investigacions Biomèdiques August Pi i Sunyer (IDIBAPS), 08036 Barcelona, Spain; 7 School of Medical Sciences, Charles Perkins Centre, Faculty of Medicine and Health, University of Sydney, Sydney, NSW 2006 Australia; 8 Present Address: Save Sight Institute, Sydney Medical School, University of Sydney, Sydney, NSW 2000 Australia; 9 Present Address: KIMS Foundation and Research Centre, KIMS Hospitals, 1-8-31/1, Minister Road, Secunderabad, Telangana 500003 India

**Keywords:** Cancer, Cell biology

## Abstract

Cholesterol is considered indispensable for cell motility, but how physiological cholesterol pools enable cells to move forward remains to be clarified. The majority of cells obtain cholesterol from the uptake of Low-Density lipoproteins (LDL) and here we demonstrate that LDL stimulates A431 squamous epithelial carcinoma and Chinese hamster ovary (CHO) cell migration and invasion. LDL also potentiated epidermal growth factor (EGF) -stimulated A431 cell migration as well as A431 invasion in 3-dimensional environments, using organotypic assays. Blocking cholesterol export from late endosomes (LE), using Niemann Pick Type C1 (NPC1) mutant cells, pharmacological NPC1 inhibition or overexpression of the annexin A6 (AnxA6) scaffold protein, compromised LDL-inducible migration and invasion. Nevertheless, NPC1 mutant cells established focal adhesions (FA) that contain activated focal adhesion kinase (pY397FAK, pY861FAK), vinculin and paxillin. Compared to controls, NPC1 mutants display increased FA numbers throughout the cell body, but lack LDL-inducible FA formation at cell edges. Strikingly, AnxA6 depletion in NPC1 mutant cells, which restores late endosomal cholesterol export in these cells, increases their cell motility and association of the cholesterol biosensor D4H with active FAK at cell edges, indicating that AnxA6-regulated transport routes contribute to cholesterol delivery to FA structures, thereby improving NPC1 mutant cell migratory behaviour.

## Introduction

Metabolic adaptations in cancer include an increased demand for membrane lipids^1^. This includes cholesterol, which supports growth but also motility, in particular the formation of focal adhesions (FA), enabling cells to move forward^2,3^. However, how cellular cholesterol pools contribute to coordinate migratory and invasive cell behaviour as well as FA assembly are not well understood.

In many tumours, upregulated de novo cholesterol synthesis is common and lowering cholesterol using statins interfered with cancer cell growth and motility^4,5^. The underlying mechanisms are complex, as statins also deplete cholesterol intermediates required for the prenylation of Ras-GTPases, thereby compromising signalling events that drive cell migration^4,5^. Importantly, for cell movement, continuous FA assembly and disassembly at the leading edge and the recruitment of focal adhesion kinase (FAK) and other signalling and structural proteins is essential^2,6^. Earlier studies identified methyl-β*-*cyclodextrin*,* which triggers a robust cholesterol depletion at the plasma membrane, to disrupt the functional integrity of FAs, suggesting cholesterol being vital for proper FA structure and assembly^2,7,8^.

Rather than synthesizing cholesterol de novo*,* most cells obtain cholesterol from Low-Density lipoprotein (LDL) uptake^3,9,10^. Once internalized, LDL-derived cholesteryl esters are hydrolyzed in late endosomes (LE)/lysosomes (Lys), to generate free cholesterol, which is then delivered to the plasma membrane. In addition, LDL-derived cholesterol is transported to the endoplasmic reticulum (ER) for acyl-CoA cholesterol acyltransferase (ACAT)-mediated re-esterification, ensuing storage as cholesteryl ester in lipid droplets^3,9,10^. This appears relevant in pancreatic ductal adenocarcinoma and other cancers, as elevated LDL receptor expression, increased LDL-cholesterol uptake and accumulation of cholesteryl esters are associated with malignant progression^11,12^. Similarly, LDL stimulated breast cancer cell migration in an ACAT-dependent manner^13^ and elevated LDL uptake led to ACAT-dependent cholesteryl ester accumulation and correlated with increased prostate cancer cell aggressiveness^14^.

Yet, the molecular players in LE/Lys conferring LDL-inducible cell motility are not well known. Several proteins, including the Niemann-Pick Type C1 (NPC1) protein, contribute to late endosomal cholesterol (LE-Chol) egress^3,9,10^. LE-Chol accumulation upon NPC1 inhibition identified dysfunctioning of soluble NSF attachment protein receptor (SNARE) proteins, including soluble N-ethylmaleimide-sensitive fusion protein 23 (SNAP23), syntaxin 4 (Stx4) and Stx6, that regulate extracellular matrix secretion and integrin recycling^9,15,16^, the latter being critical for FA assembly and cell motility^6,16^. In line with this, NPC1 depletion in cervical cancer cells inhibited cell proliferation and migration^17^. Lysosomotropic compounds^18^ and the anti-histamine astemizole^19^ were also found to block LE-Chol egress and retarded melanoma tumour growth and cell migration.

In addition, upregulation of annexin A6 (AnxA6), an annexin regulating endo-/exocytic pathways, cholesterol transport and assembly of signalling complexes^20–22^, also caused LE-Chol accumulation^23^. Alike NPC1 deficiency, AnxA6 upregulation was accompanied by cholesterol depletion in other cellular sites, including the plasma membrane, Golgi apparatus and recycling endosomes. This cellular cholesterol imbalance triggered the mislocalization and dysfunction of SNAP23, Stx4 and Stx6, interfering with cell surface delivery of integrins, extracellular matrix proteins, as well as cholesterol, being detrimental for cell migration and invasion^15,24,25^.

Annexins, including AnxA6, bind to membranes in a Ca^2+^-dependent manner, but LDL loading or loss of NPC1 function led to the association of significant AnxA6 amounts with cholesterol-rich LE/Lys^26,27^. This enabled the AnxA6-mediated recruitment of the Rab7-GTPase activating protein (Rab7-GAP) TBC1D15 to downregulate Rab7 activity, which interfered with LE-Chol egress^28^. Strikingly, AnxA6 depletion in NPC1 mutant cells upregulated Rab7 activity, rescuing LE-Chol export for cholesteryl ester storage in lipid droplets via transport routes involving the StAR-related lipid transfer domain-3 (StARD3) protein^28,29^. These findings indicated that AnxA6 controls LE-Chol export routes that operate in the absence of NPC1^28,29^.

Here we demonstrate that pharmacological or genetic NPC1 inhibition as well as AnxA6 overexpression strongly reduced the ability of LDL to stimulate A431 cell migration and invasion or to potentiate epidermal growth factor (EGF)-inducible A431 cell migration. Interestingly, high FA numbers in NPC1 mutant cells identify their ability to overcome reduced cholesterol levels at the plasma membrane in order to establish FA structures. In addition, FA redistribution in NPC1 mutant cells occurred, with less FAs at the cell edges and more FAs across the cell body. Strikingly, AnxA6 depletion in NPC1 mutant cells was associated with increased motility, and elevated association of cholesterol with FA at cell edges, indicating that AnxA6 deficiency promotes NPC1-independent cholesterol transport routes to FA. The contribution of the cholesterol transport machinery in LE/Lys to increase cancer growth and motility in an LDL-rich microenvironment is discussed.

## Results

### AnxA6 overexpression and pharmacological NPC1 inhibition reduce A431 cell migration

**Figure 1:**
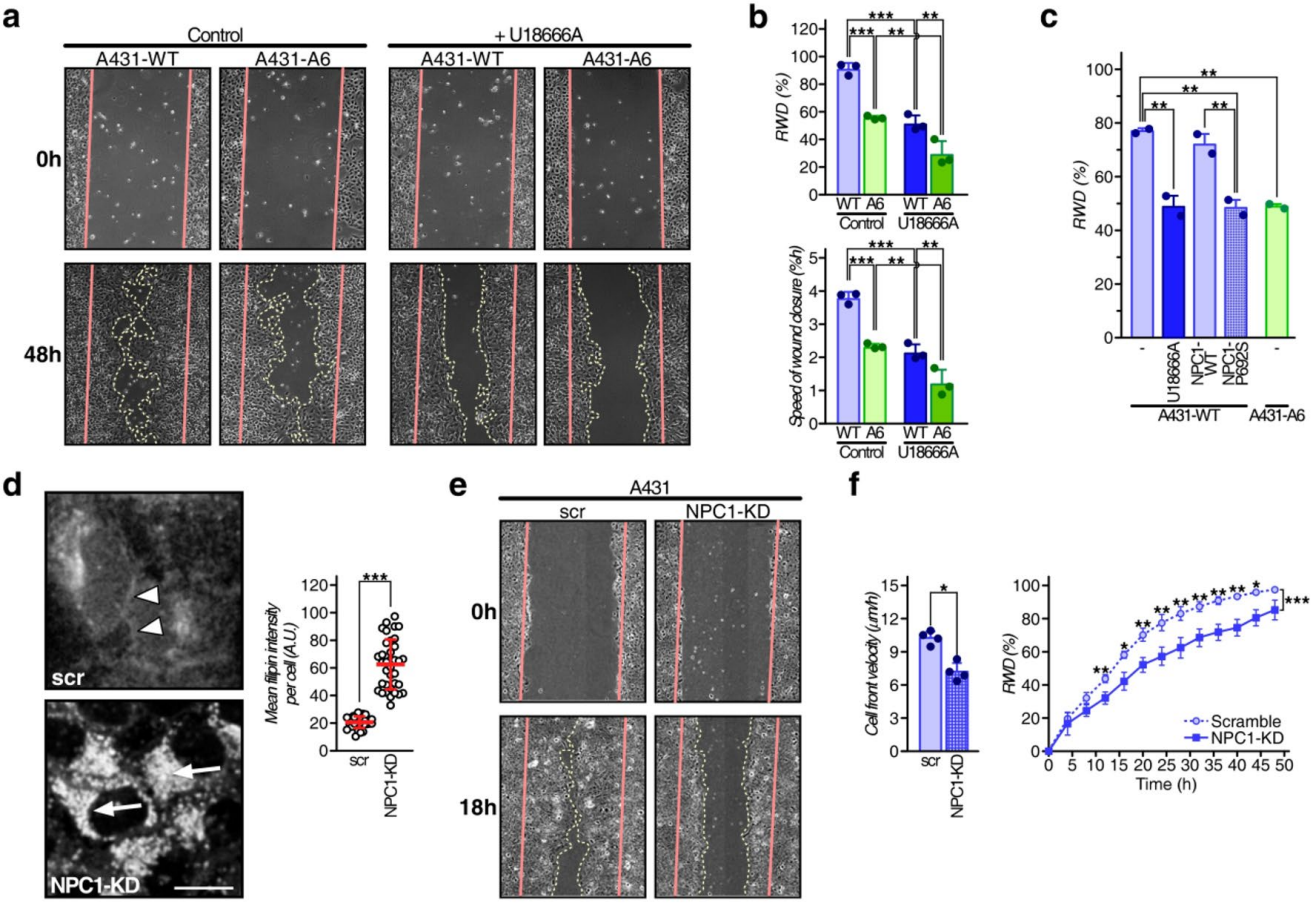
AnxA6 overexpression and pharmacological NPC1 inhibition reduce A431 cell migration. (**a**,**b**) A431-WT and A431-A6 cells were treated ± U18666A (4 µg/ml) and then wound healing assays were performed. Representative images at t = 0 and 24 h are shown. The relative wound density (RWD %) and speed of wound closure (%/h) from 3 independent experiments with triplicate samples (mean ± SD) is shown. (**c**) A431-WT treated ± U18666A (4 µg/ml), ectopically expressing NPC1 wildtype (NPC1-WT) or mutant (NPC1-P692S), and A431-A6 cells were grown until 90% confluency and then wound healing assays were performed. The RWD (%) at t = 18 h from 2 independent experiments with duplicate samples (mean ± SD) was calculated. (**d**) A431-WT stably expressing scrambled shRNA (scr) or a combination of four shRNAs (1–4) targeting NPC1 (NPC1-KD) were fixed and stained with filipin. Arrowheads indicate filipin staining at the plasma membrane of control cells. Arrows point at cholesterol accumulation in perinuclear compartments of NPC1-depleted A431-WT cells. The mean filipin intensity per cell (a.u.) in 32 control (scr) and 36 NPC1-KD cells was quantified (mean ± SD). Bar is 10 µm. (**e**–**f**) Wound healing assays with A431-WT stably expressing scrambled shRNA (scr) or NPC1-depleted A431-WT cells using the IncuCyte were performed. Representative images at t = 0 and 18 h are shown (**e**). Cell migration was monitored at 2 h intervals and RWD (%) and cell velocity (µm/h) from 2 independent experiments with quadruple (left panel) or quintuple samples (right panel) was calculated (mean ± SD). The mean of individual experiments (**b**,**c**) and individual data points (**f**, left panel) is indicated by dot points in each bar graph. *p < 0.05, **p < 0.01, ***p < 0.001; two-way ANOVA with Tukey’s post-hoc test (**b**,**f**), one-way ANOVA with Tukey’s post-hoc test (**c**) and Mann–Whitney two-tailed test (**f**).

NPC1 deficiency or AnxA6 overexpression in Chinese hamster ovary (CHO) and A431 squamous epithelial carcinoma cells reduced cell migration and invasion in 2- and 3-dimensional environments^16,24^. To validate these findings, cell migration of wildtype A431 cells (A431-WT), which lack endogenous AnxA6, and the well-characterized A431 cell line stably expressing AnxA6 (A431-A6)^30,31^ in the presence of the pharmacological NPC1 inhibitor U18666A was analysed (Fig. 1a,b).

A431-WT and A431-A6 cells were grown in serum-containing media (10% FCS) and treated ± U18666A (4 μg/ml) overnight. Then scratch assays were performed and alike previous studies^24^, in the absence of U18666A, A431-WT showed ~ 40% greater wound closure (relative wound density %; RWD %) and speed of wound closure (%/h) compared to A431-A6 cells (Fig. 1a,b). Under these lipid-rich conditions, U18666A-induced LE-Chol accumulation^16,28^ in A431-WT was accompanied by a strongly reduced wound closure and cell velocity. In A431-A6 cells, which already exhibit an NPC1-like phenotype in serum-containing media^23^, this inhibitory effect of U18666A on cell migration was less pronounced (Fig. 1b).

Likewise, ectopic expression of the loss-of-function NPC1 mutant P692S, which cannot bind cholesterol and inhibits LE-Chol export^16,32^, inhibited A431 cell migration similar to U18666A treatment or AnxA6 overexpression (Fig. 1c). Stable shRNA-mediated NPC1 knockdown (≥ 35%; clone 1–4) in A431 cells (Suppl. Figure 1a), which caused substantial LE-Chol accumulation in serum-containing media (see arrows and mean filipin intensity per cell in Fig. 1d), also significantly reduced wound closure and cell velocity (Fig. 1e,f).

### LDL-inducible A431 cell migration is compromised by AnxA6 overexpression or NPC1 inhibition

**Figure 2:**
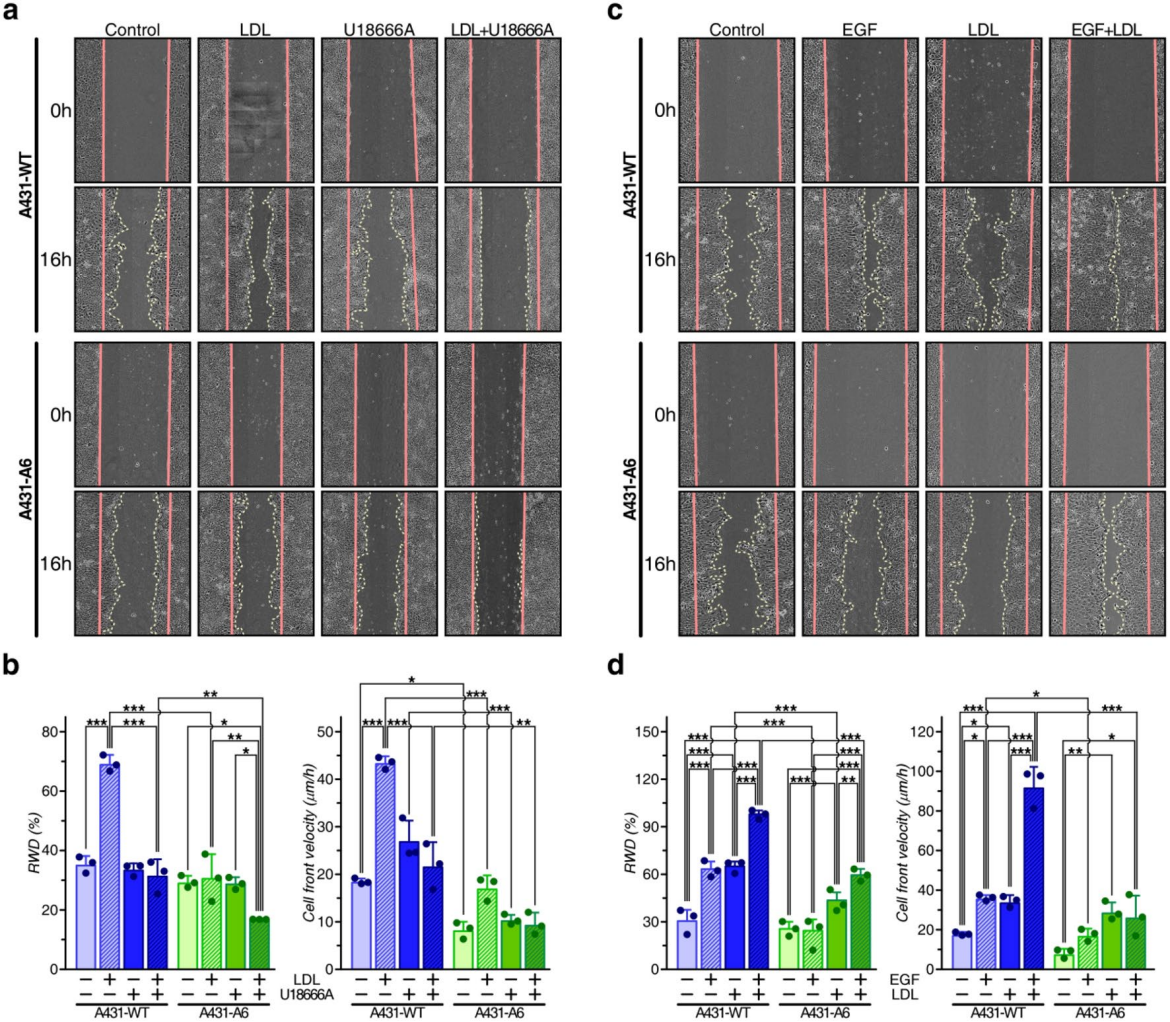
AnxA6 overexpression and NPC1 inhibition compromise LDL-inducible cell migration. (**a**,**b**) Wound healing assays with A431-WT and A431-A6 cells grown in 10% LPDS-containing media together with LDL (50 µg/ml), U18666A (4 µg/ml), or both using the IncuCyte were performed. Representative images at t = 0 and 16 h are shown (**a**). RWD (%) and cell velocity (µm/h) from 3 independent experiments with triplicate samples (mean ± SD) was calculated (**b**). (**c**,**d**) A431-WT and A431-A6 cells were grown in LPDS-containing media ± LDL (50 µg/ml) and EGF (10 ng/ml) as indicated and cell migration was monitored. Representative images at t = 0 and 14 h are shown (**c**). RWD (%) and cell velocity (µm/h) from 3 independent experiments with triplicate samples (mean ± SD) was calculated (**d**). For each individual experiment shown in (**b**,**d**), the mean is indicated by dot points in each bar graph. *p < 0.05, **p < 0.01, ***p < 0.001; two-way ANOVA with Tukey’s post-hoc test.

To examine if LDL-derived cholesterol could induce cell migration, A431-WT and A431-A6 cells were pre-incubated in 10% lipoprotein-deficient serum (10% LPDS)-containing media to lower cellular cholesterol in LE^10,28^. Then cells were loaded with LDL (50 µg/ml) ± U18666A (4 μg/ml) for additional 24 h and lipids were extracted to determine cellular cholesterol levels (see “Methods” section) and confirm LDL-cholesterol loading (Suppl. Figure 1b). When cells were grown without LDL in lipoprotein-deficient media, U18666A alone did not significantly increase cellular cholesterol levels compared to controls. As expected, LDL increased cellular cholesterol levels, which was elevated even further upon pharmacological NPC1 inhibition, in both cell lines (Suppl. Figure 1b). Then wound healing assays ± LDL, U18666A or both were performed (Fig. 2a,b). In lipid-depleted A431-WT cells, which contain only minor amounts of LE-Chol^28^, U18666A did not significantly impact on cell migration. In contrast, LDL strongly stimulated A431-WT wound closure and cell velocity (~ 2–2.5 -fold), which was effectively blocked by co-incubation with U18666A. In line with previous findings^24^, cell migration of lipid-depleted A431-A6 cells was significantly reduced compared to A431-WT cells. Yet, in striking contrast to A431-WT cells, LDL did not stimulate A431-A6 wound closure and had a much smaller impact on cell velocity. Thus, NPC1 inhibition and AnxA6 overexpression, which both inhibit LDL-derived LE-Chol egress^9,23,28^, interfere with the ability of LDL to stimulate A431 cell migration.

A431-WT cells express large amounts (1–3 × 10^6^/cell) of epidermal growth factor receptor (EGFR). In these cells, AnxA6 downregulates EGFR activity and EGF-induced cell growth and migration^31,33^. To examine if LDL loading could potentiate EGF-inducible cell migration, A431-WT and A431-A6 cells were loaded ± LDL as described above, and wound healing assays ± EGF (10 ng/ml) were performed (Fig. 2c,d). As shown previously^33^, A431-WT cells displayed a much greater EGF-inducible wound closure (RWD %) and cell velocity (µm/h) compared to A431-A6 cells (Fig. 2d). As above, LDL strongly stimulated A431-WT, and to a smaller extend A431-A6 cell migration. Strikingly, EGF and LDL co-incubation greatly stimulated wound closure and cell velocity in A431-WT cells. Hence, growth factors and lipoproteins can cooperate to stimulate A431 cell motility. This was much less pronounced in AnxA6-expressing A431 cells, probably due to the inhibitory role of AnxA6 in EGFR signalling^31,33^ and LE-Chol export^23,24,28^.

### Annexin A6 inhibits LDL-inducible invasive properties of A431 cells

**Figure 3:**
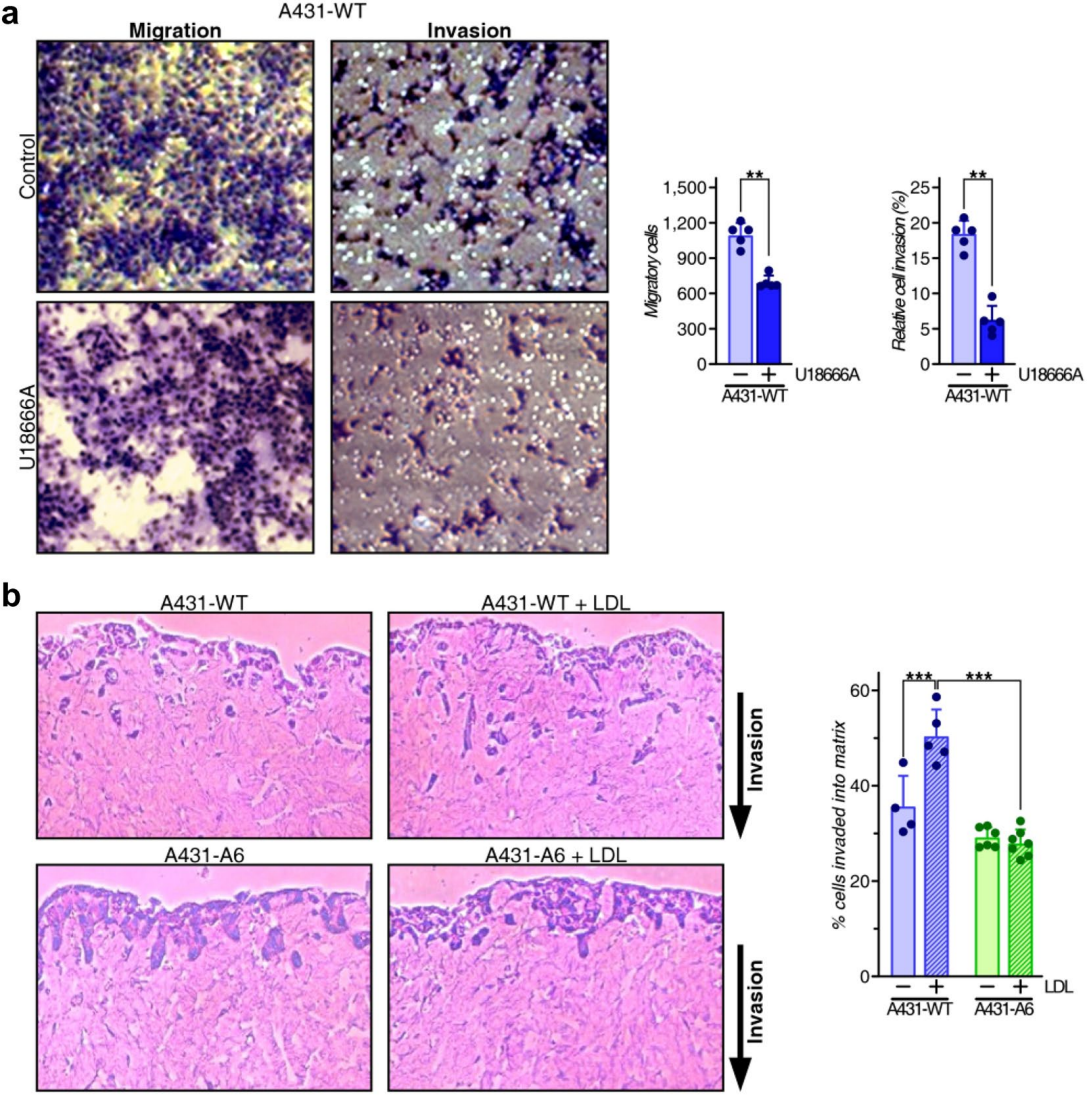
NPC1 inhibition and AnxA6 overexpression inhibit LDL-inducible A431 cell invasion. (**a**) Transwell migration and invasion of A431-WT cells treated ± U18666A (4 µg/ml). Representative images of migrating and invading cells from five fields per condition of a representative experiment (n = 2) were quantified and relative invasion (%) was calculated (mean ± SD). Individual data points from the five fields are indicated by dot points in each bar graph. (**b**) A431-WT and A431-A6 cells on three-dimensional matrices of rat tail collagen were grown in 10% LPDS-containing media ± LDL (50 μg/ml) fixed, and stained with hematoxylin and eosin. The relative invasion (%) was calculated as the percentage of cells that invaded beyond ≈30 μm as a percentage of total cells in the assay. Representative images from 2 independent experiments are shown. The mean ± SD from 4–6 images per condition is given. Individual data points are indicated by dot points in each bar graph. *p < 0.05, **p < 0.01, ***p < 0.001; Mann–Whitney two-tailed test (**a**), and two-way ANOVA with Tukey’s post-hoc test (**b**).

We then examined if pharmacological NPC1 inhibition interfered with cell invasion using transwell matrigel invasion chambers (Fig. 3a). A431-WT cells were seeded in the upper chamber in serum-free medium and incubated ± U18666A (4 μg/ml). The lower chamber contained 10% FCS as chemoattractant and cells that invaded through the coated membrane inserts into the lower chamber were determined. Cell migration with uncoated inserts served as control. In line with findings described above (Figs. 1, 2), U18666A strongly reduced migration of A431-WT cells through uncoated inserts, but also decreased the invasive capacity of A431-WT cells across coated inserts into the lower wells (Fig. 3a).

Next, in three-dimensional organotypic matrices that more closely recapitulate a tumour stromal environment *in vivo*^16,24,25^, LDL significantly stimulated A431-WT invasion into organotypic matrices over a 14-day period (Fig. 3b). In contrast, A431-A6 cells showed a reduced ability to invade these matrices^30^, which was not stimulated by LDL. Hence, elevated LDL levels in the tumour microenvironment may improve the invading properties of cancer cells. Moreover, the inhibitory role of AnxA6 in the cellular distribution of LDL-derived cholesterol^28^ most likely compromised LDL-inducible invasive properties of A431 cells.

### LDL promotes cell migration in CHO-WT, but not NPC1 mutant CHO cells

**Figure 4:**
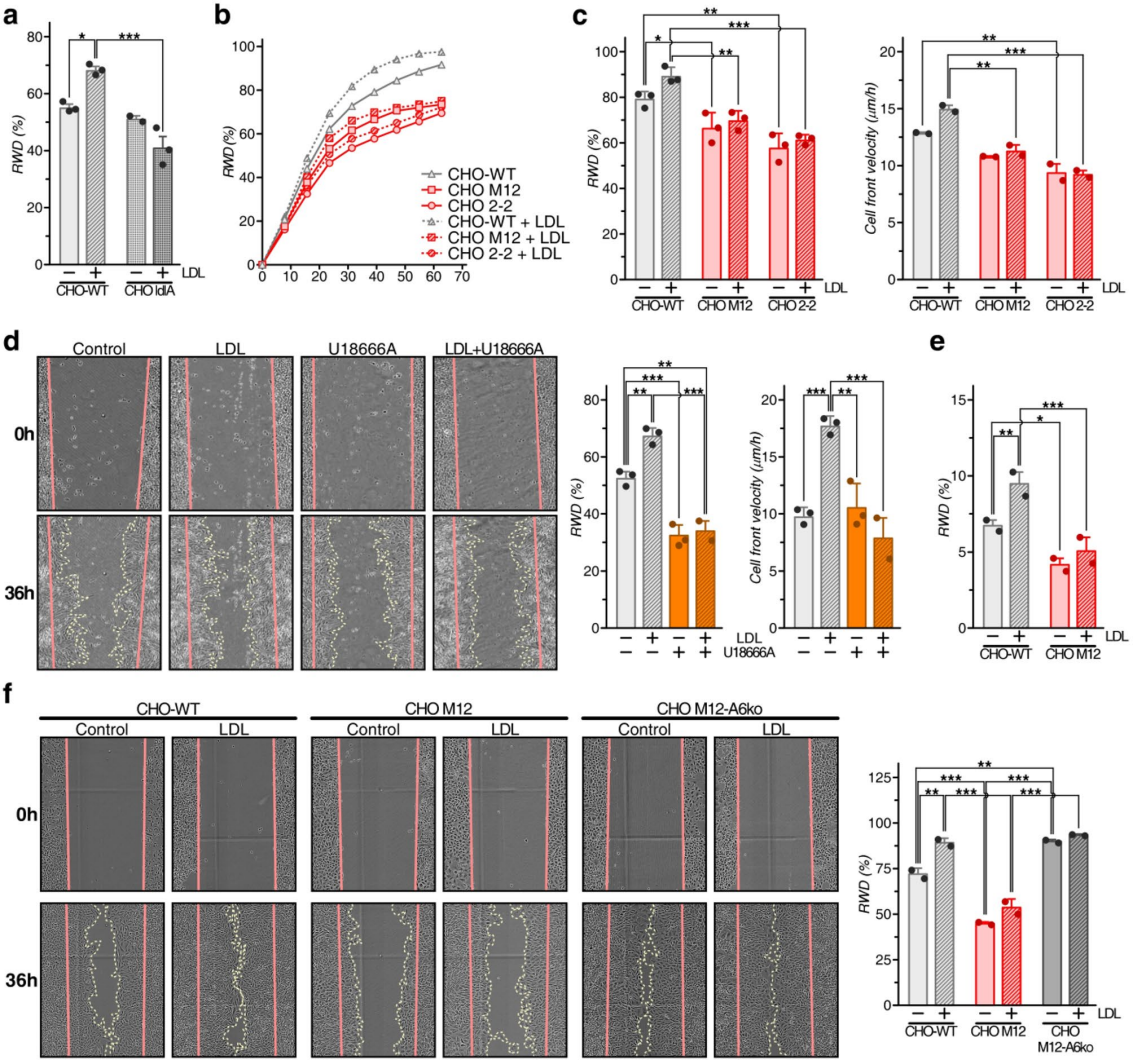
LDL promotes cell migration in CHO-WT, but not NPC1 mutant CHO cells. (**a**) Wound healing assays of CHO-WT, CHO ldlA and (**b**) CHO-WT, CHO M12 and CHO 2–2 cells grown in 10% LPDS-containing media ± LDL (50 µg/ml). Cell migration was monitored for 0–60 h. RWD (%) from 3 independent experiments with triplicate samples (mean ± SD) at 44 h (**a**) and for 0–60 h (**b**) for a representative experiment is shown. (**c**) RWD (%) and cell velocity (µm/h) at 40 h from 2–3 independent experiments with triplicate samples (mean ± SD) was calculated. (**d**) Wound healing assays with CHO-WT cells grown in 10% LPDS-containing media ± LDL (50 µg/ml), U18666A (4 µg/ml), or both as indicated. Cell migration was monitored and representative images at t = 0 and 48 h are shown. RWD (%) and cell velocity (µm/h) from 3 independent experiments with triplicate samples (mean ± SD) was calculated. (**e**) Scratch assays with CHO-WT and CHO M12 cells grown in 10% LPDS-containing media and loaded ± LDL (50 μg/ml) for 2 h prior the scratch. The media and cell debris were removed and cell migration in the absence of LDL was monitored for 8 h. RWD (%) from 2 independent experiments with triplicate samples (mean ± SD) was calculated. (**f**) CHO-WT, CHO M12 and CHO M12-A6ko cells were incubated ± LDL as described in (**d**) and RWD (%) at 40 h from 2 independent experiments with triplicate samples (mean ± SD) was calculated. The mean of each individual experiment (**a**,**c**–**f**) is indicated by dot points in each bar graph. *p < 0.05, **p < 0.01, ***p < 0.001; two-way ANOVA with Tukey’s post-hoc test (**a**,**c**,**e**,**f**), one-way ANOVA with Tukey’s post-hoc test (**d**).

To strengthen LDL endocytosis as the underlying mechanism stimulating cell migration, we next compared wildtype CHO cells (CHO-WT) and a CHO line lacking the LDL receptor (CHO ldlA)^34^. CHO-WT and CHO ldlA cells were pre-incubated in lipoprotein-deficient media as above, scratched, and then cell migration ± LDL was monitored (Fig. 4a). LDL improved wound closure of CHO-WT (≥ 20%), but not CHO ldlA cells, at later time points (≥ 32 h), supporting LDL endocytosis to induce cell migration.

Next, CHO-WT and CHO cell lines lacking NPC1 (CHO M12, CHO 2–2) were pre-incubated in 10% LPDS-containing media and then wound closure ± LDL was monitored (Fig. 4b,c). As shown previously^16^, NPC1 mutant cells displayed significantly reduced wound closure and cell velocity compared to wildtype controls. Alike A431-WT cells (Fig. 2b,c), LDL stimulated CHO-WT, but not CHO M12 and CHO 2–2 cell motility at later time points (Fig. 4b,c). Likewise, pharmacological NPC1 inhibition effectively blocked LDL-induced CHO-WT wound closure and cell velocity (Fig. 4d).

To reinforce export of LDL-derived Chol from LE to support cell migration, pulse-chase experiments were performed. CHO-WT and CHO M12 cells were grown in LPDS-supplemented media and then treated ± LDL for 2 h to load the LE/Lys compartment^23^. Then the LDL-containing media was removed and cell motility in lipoprotein-deficient media was monitored (Fig. 4e). Indeed, LDL pre-loading stimulated CHO-WT, but not CHO M12 cell migration, indicating that LDL-derived LE-Chol egress to other cellular sites stimulates cell motility.

AnxA6 depletion restored LE-Chol egress in CHO M12 cells^28^. To examine if this could change migratory behaviour, CHO M12 and CHO M12-A6ko cells were pre-incubated in lipoprotein-deficient media, and then wound closure ± LDL was monitored (Fig. 4f). Strikingly, CHO M12-A6ko cells showed a strongly increased ability to close the wound compared to CHO M12 cells already in the absence of LDL, indicating that AnxA6 deficiency could overcome dysfunctional and possibly cholesterol-dependent events in NPC1 mutant cells that govern cell migration.

### NPC1 deficiency in CHO cells increases FA number and alters FA distribution

**Figure 5:**
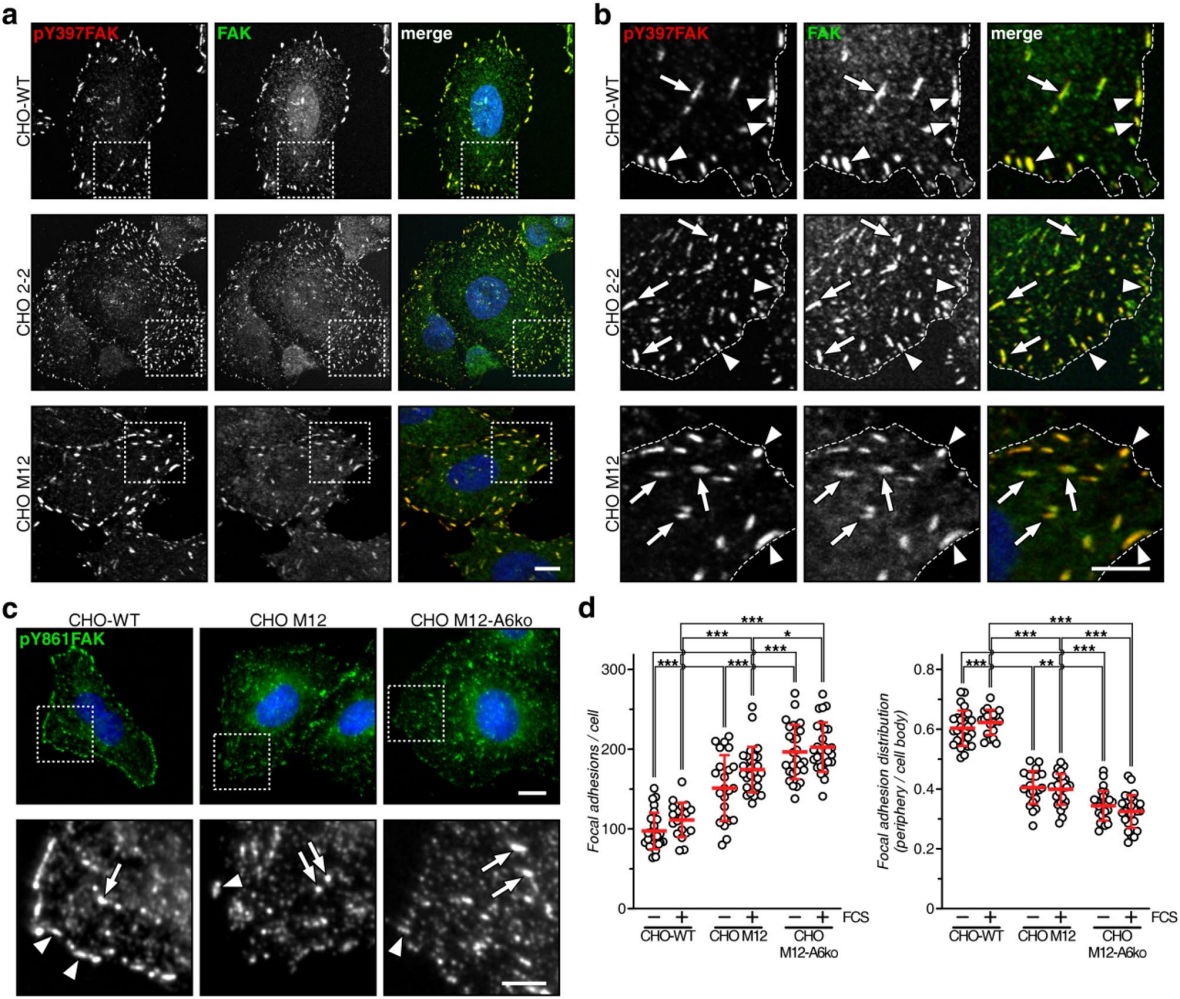
NPC1 deficiency in CHO cells alters the number and distribution of focal adhesions. (**a**,**b**) CHO-WT, CHO M12 and CHO 2–2 cells were stained for total FAK (green), phosphorylated FAK (pY397FAK, red) and nuclei (DAPI, blue) as indicated. The merged images and enlarged regions of interest (**b**) are shown. (**c**) CHO-WT, CHO M12 and CHO M12-A6ko cells were stained for phosphorylated FAK (pY861FAK, green), and nuclei (DAPI, blue) as indicated. Enlarged regions of interest (lower panel) are shown. Large FA complexes at the cell edge (arrowheads) and across the cell body (arrows) are indicated. Bar is 10 µm (**a**,**c**) and 5 µm for enlarged insets (**b**,**c**), respectively. (**d**) Focal adhesions (> 0.25 µm^2^) per cell and the ratio of focal adhesions at the cell edge vs. cell body in starved and serum-activated (20% FCS, 45 min) cells were quantified. 20–27 cells per condition and cell line were counted. The mean ± SD is given. *p < 0.05, **p < 0.01, ***p < 0.001; two-way ANOVA with Tukey’s post-hoc test).

Reduced cell motility and plasma membrane cholesterol levels in NPC1 mutant cells (Figs. 1c–f, 4b,c,e,f)^9,16,23^ prompted us to compare FA distribution in CHO-WT, M12 and 2–2 cells (Fig. 5a,b). Therefore, cells were grown in serum-containing media, fixed and stained for total and phosphorylated (pY397) FAK, which transmits signals from cell surface receptors to regulate FA (dis-)assembly for cell movement, with pY397FAK commonly serving as FA marker^2^.

In CHO-WT cells, pY397FAK colocalized with total FAK in often large, macroscopic structures at the cell edges (Fig. 5b, see arrowheads in enlarged insets). Interestingly, both NPC1 mutant CHO cell lines contained plenty of large and pY397FAK-positive complexes at the cell edges, but also across the cell body (see arrows in Fig. 5b). Hence, despite their cellular cholesterol imbalance^9,16,23^, NPC1 mutant cells can establish pY397FAK-containing macroscopic FA structures, which appear to be distributed differently compared to control cells.

### AnxA6-depleted NPC1 mutant cells display elevated FA numbers and altered FA distribution

FA formation requires the recruitment of Src kinase to pY397FAK, followed by Src-mediated Y861FAK phosphorylation^35^. We therefore compared pY861FAK staining in CHO-WT, CHO M12 and AnxA6-depleted CHO M12 (CHO M12-A6ko) cells (Fig. 5c). Similar to the pY397FAK staining patterns (Fig. 5a,b), CHO-WT, M12 and M12-A6ko cells contained large numbers of pY861FAK-positive complexes. To address their ability to respond to extracellular stimuli that promote FA formation, cells were starved overnight, serum-stimulated (20% fetal calf serum; 20% FCS) for 45 min, fixed and stained with anti-pY861FAK. Then FA complexes (≥ 0.25 µm^2^) per cell were quantified (Fig. 5d). A trend of increased FA numbers upon serum stimulation was observed in all three cell lines, indicating that Src-dependent FAK activation and FA assembly can still occur despite a cellular cholesterol imbalance in NPC1 mutant cells. FA numbers were strongly increased in CHO M12 and M12-A6ko compared to controls (left panel in 5d). Moreover, alike the p397FAK staining (Fig. 5a,b), CHO-WT cells showed a higher percentage of pY861FAK-containing complexes at the cell edges, while these structures were distributed more throughout the cell body in CHO M12 and M12-A6ko cells. This observation appeared independent of FA size, as the number of large (≥ 1 µm^2^), medium (0.5–1 µm^2^) and small (0.25–0.5 µm^2^) FA complexes per cell was strongly elevated overall, at the cell edge and throughout the cell body, in CHO M12 and CHO M12-A6ko cells (Suppl. Figure 2). Hence, NPC1 deficiency in CHO cells appears associated with an increased and altered distribution of FA complexes. AnxA6 depletion in CHO M12 cells further increased total FA numbers, in particular throughout the cell body, indicating a role for AnxA6 in the dynamics of FA (dis-)assembly in NPC1 mutant cells.

### Distribution of FA marker proteins vinculin and paxillin in CHO M12 and CHO M12-A6ko cells

**Figure 6:**
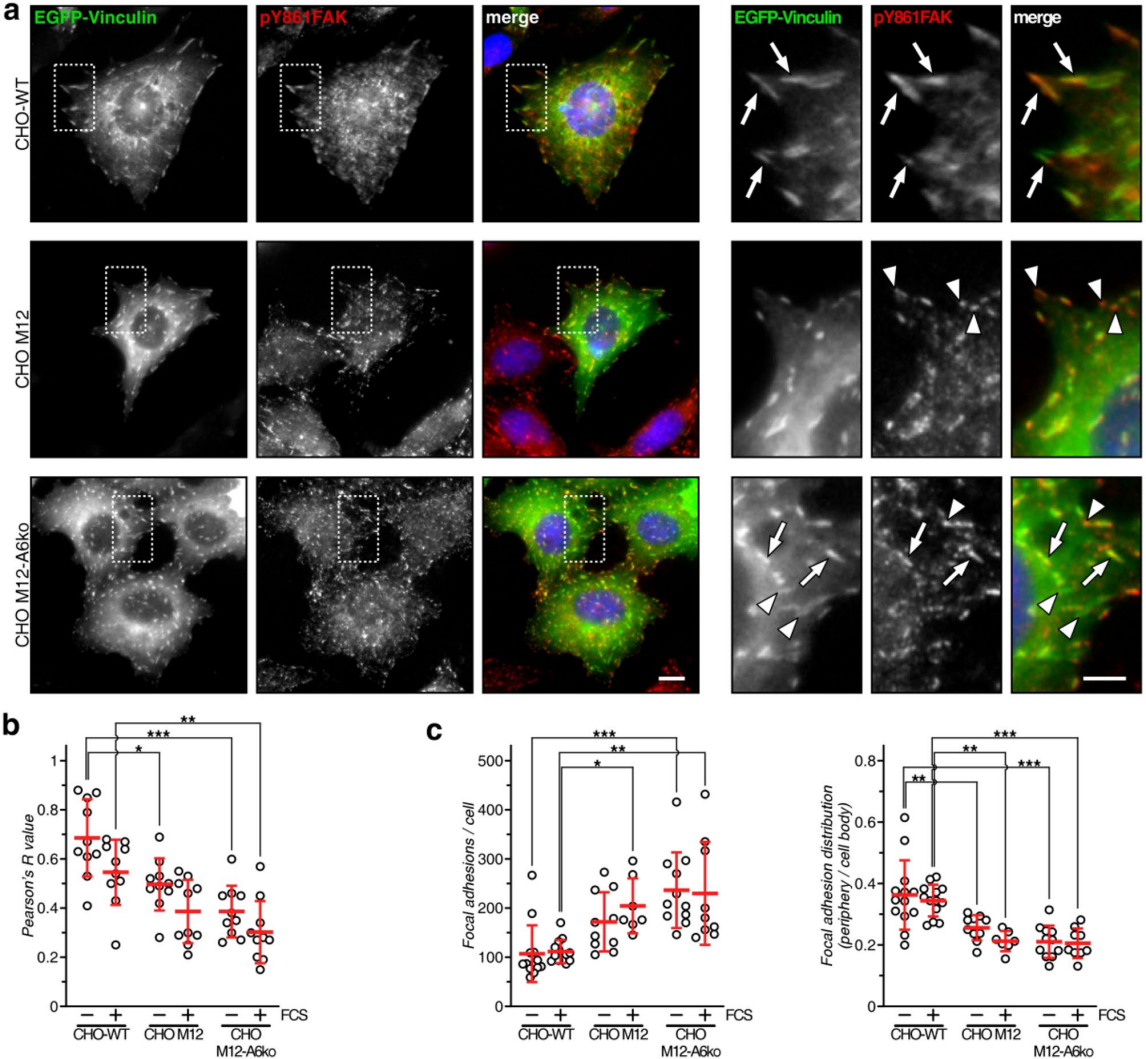
Distribution of FA marker vinculin in CHO M12 and CHO M12-A6ko cells. (**a**) CHO-WT, CHO M12 and CHO M12-A6ko cells ectopically expressing EGFP-vinculin were serum-stimulated (20% FCS) and stained for phosphorylated FAK (pY861FAK, red) as indicated. The merged images and enlarged regions of interest are shown. Arrows indicate colocalization of EGFP-vinculin and pY861FAK in CHO-WT cells. Arrowheads point at pY861FAK in CHO M12 or EGFP-vinculin staining in CHO M12-A6ko cells, respectively. Bar is 10 µm and 5 µm for enlarged insets. (**b**) Colocalization of EGFR-vinculin and pY891FAK was quantified (Pearson’s R value; 20–30 cells per condition and cell line were quantified. (**c**) Focal adhesions (> 0.25 µm^2^) per cell (left panel) and the ratio of focal adhesions at the cell edge vs. cell body (right panel) was quantified. 7–13 cells per condition and cell line were counted. The mean ± SD is given. *p < 0.05, **p < 0.01, ***p < 0.001; two-way ANOVA with Tukey’s post-hoc test.

FA are complex, heterogeneous and multiprotein structures^2,6,35^. Therefore, we compared the cellular distribution of pY861FAK and vinculin, a structural protein associated with FA^6^. CHO-WT, CHO M12 and CHO M12-A6ko cells were transfected with EGFP-vinculin, starved overnight, serum-stimulated (20% FCS) and stained with anti-pY861FAK (Fig. 6a). In CHO-WT cells, vinculin often colocalized with pY861FAK in large structures at the cell edges and throughout the cell body (see arrows in enlarged inset). EGFP-vinculin/pY861FAK colocalization was significantly reduced in CHO M12 and even more so in CHO M12-A6ko cells (Fig. 6b; see also arrowheads in enlarged insets in 6a), implying NPC1 deficiency to manifest in a more heterogenous population of FA complexes. Upon serum stimulation, EGFP-vinculin/pY861FAK colocalization was reduced in all cell lines, indicating similar changes in FA structure and protein composition. Alike pY861FAK staining patterns (Fig. 5d), EGFP-vinculin distribution revealed increased FA numbers per cell (Fig. 6c, left panel) and in the cell body (Fig. 6c, right panel) of CHO M12 and even more so in CHO M12 A6ko cells, compared to controls. Thus, FA structures in NPC1 mutant cells appear more heterogenous and distributed more across the cell body, in particular upon AnxA6 depletion, compared to control cells.

Alike vinculin, ectopically expressed EGFP-paxillin, another protein associated with FA, often colocalized with pY861FAK at cell edges and throughout the cell body (Suppl. Figure 3, see arrows in enlarged inset) in serum-stimulated WT, and slightly less in CHO M12 and CHO M12-A6ko cells. Similar to the FA analysis based on pY861FAK and EGFP-vinculin (Figs. 5d, 6c), EGFP-paxillin staining showed increased FA numbers throughout the cell body in CHO M12 and CHO M12-A6ko cells compared to controls. Taken together, despite substantially reduced plasma membrane cholesterol levels^9,16^, NPC1 mutant cells can create multifactorial FA complexes. Loss of AnxA6 scaffolding roles in LE important for LE-Chol transfer^28,29^, or signal complex formation at the plasma membrane^20,25,30,31^, seem to influence the number, distribution and protein composition of FAs in NPC1 mutant cells.

### LDL stimulates FA formation at the cell edges of WT, but not NPC1-deficient cells

**Figure 7:**
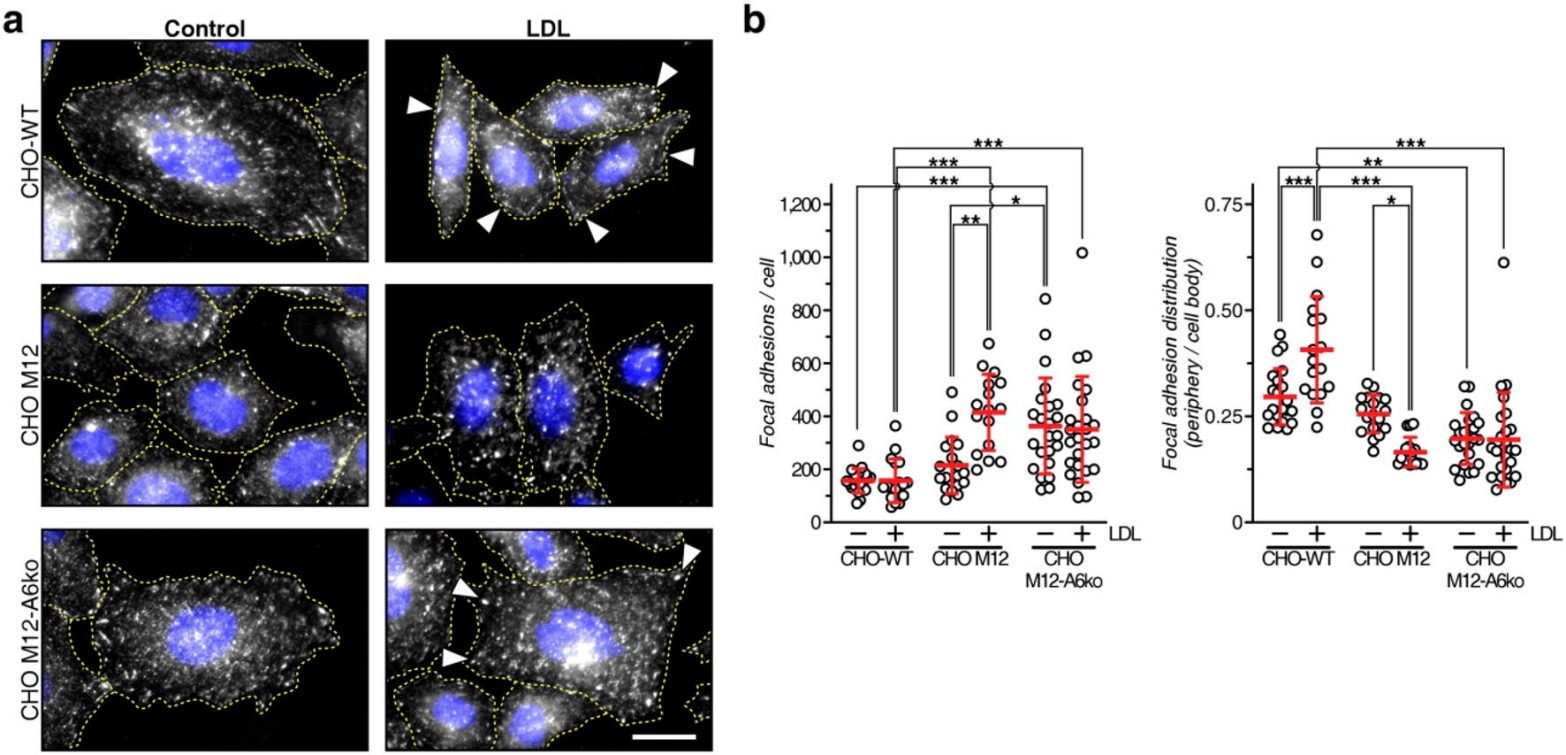
LDL stimulates FA formation at the cell edges of WT, but not NPC1-deficient cells. (**a**) CHO-WT, CHO M12 and CHO M12-A6ko cells grown in 10% LPDS-containing media ± LDL (50 μg/ml) were immunolabeled with anti-pY861FAK. Arrowheads point at macroscopic pY861FAK-positive structures at the cell edge in CHO-WT and CHO M12-A6ko cells. Bar is 20 µm. (**b**) Focal adhesions (> 0.25 µm^2^) per cell (left panel) and the ratio of focal adhesions at the cell edge vs. cell body (right panel) were quantified. 16–21 cells per condition and cell line were counted. The mean ± SD is given. *p < 0.05, **p < 0.01, ***p < 0.001; two-way ANOVA with Tukey’s post-hoc test.

Next, CHO-WT, CHO M12 and CHO M12-A6ko cells treated ± LDL for 4 h, which is sufficient to load the LE/Lys compartment and deliver LDL-derived cholesterol to the plasma membrane^23,36^, were immunolabeled with anti-pY861FAK, and FA number, distribution and size was quantified (Fig. 7a,b, Suppl. Figure 4). Alike cells grown in full serum and/or after serum activation (Figs. 5, 6, Suppl. Figure 3), total FA numbers in CHO-WT grown in lipid-depleted media were lower compared to M12 and even more so when compared to M12-A6ko cells (Fig. 7b, left panel). As described above (Figs. 5, 6), CHO-WT cells displayed relatively more FA at cell edges compared to M12 and M12-A6ko cells (right panel).

The presence of LDL did not raise total FA numbers in CHO-WT cells, yet increased FA numbers at the cell edge compared to the cell body (Fig. 7b, right panel), indicating LDL to stimulate FA turnover for cell movement. LDL also appeared to elevate the number of large FA (≥ 1 µm^2^) in CHO-WT, but also M12 and M12-A6ko cells, yet this was not significant (Suppl. Figure 4). While there was a trend of LDL increasing total FAs in CHO M12 cells, the relative number of FAs at the cell edge in these cells was significantly reduced (Fig. 7b, right panel). This supports findings described above (Figs. 1, 2, 3), that LDL could not stimulate cell migration and invasions when LE-Chol egress is blocked. LDL also increased total FA numbers in CHO M12-A6ko cells (Fig. 7b, left panel), yet the ratio of peripheral/cell body FAs slightly increased in the presence of LDL (right panel), indicating AnxA6 depletion to partially restore kinetics of FA turnover in NPC1 deficient cells, which could contribute to increase CHO M12-A6ko cell motility (Fig. 4f). Indeed, although not significant, we often observed prominent FA staining at cell edges of LDL-incubated CHO M12-A6ko cells (see arrowheads in Fig. 7a).

### AnxA6-deficient NPC1 mutant cells display increased association of cholesterol with FA structures

**Figure 8:**
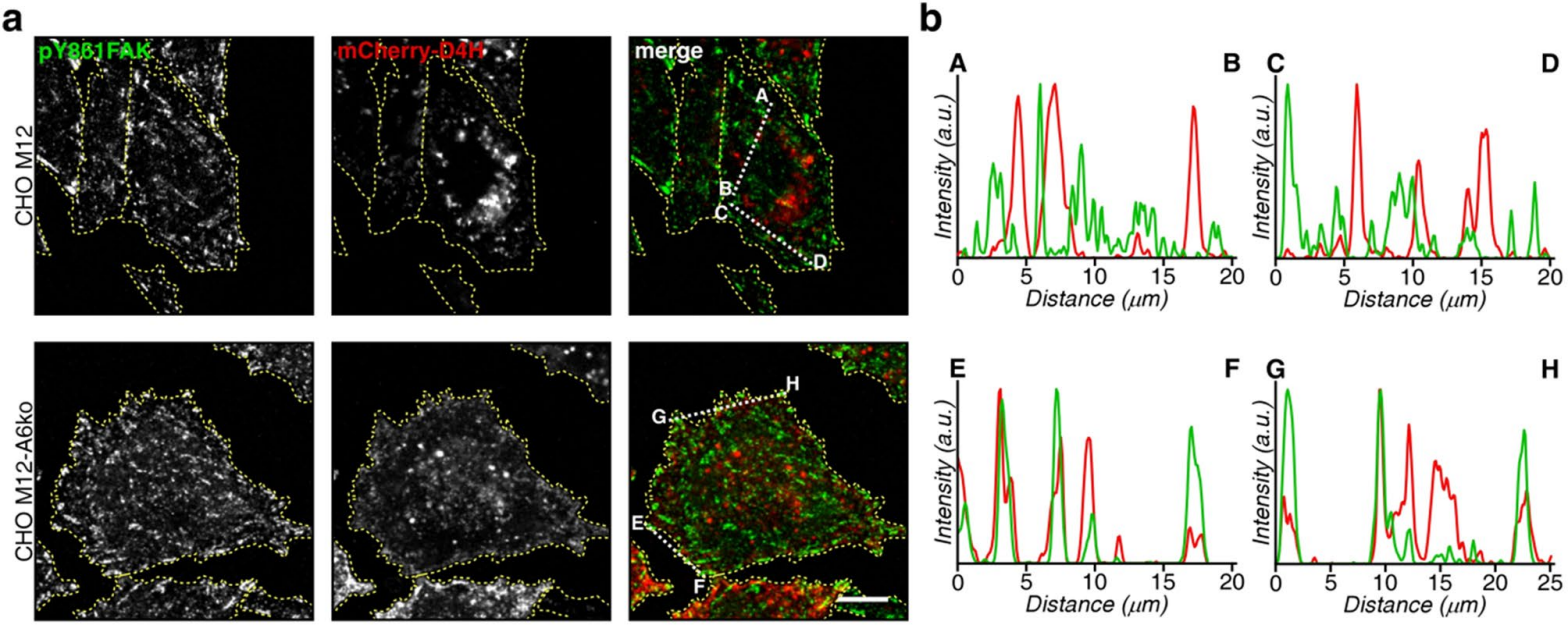
AnxA6-deficient NPC1 mutant cells display increased association of cholesterol with FA structures. (**a**) CHO M12 and CHO M12-A6ko cells ectopically expressing the cholesterol biosensor mCherry-D4H (red) were serum-stimulated (20% FCS) and stained for phosphorylated FAK (pY861FAK, green) as indicated. The merged images are shown. In CHO M12 cells, different distributions of D4H and pY861-FAK are common (see line profiles A–B, C–D, in **b**, top panel), respectively. In CHO M12-A6ko cells, D4H and pY861FAK often colocalize at cell edges (see line profiles E–F, G–H in **b**, bottom panel). Bar is 10 µm. (**b**) The line profiles of fluorescence intensities (a.u.) of pY861FAK (green) and mCherry–D4H (red) in CHO M12 (A–B, C–D, 0–20 μm, top panel) and CHO M12-A6ko cells (E–F, 0–20 μm and G–H, 0–25 μm, bottom panel) are shown.

LDL-derived cholesterol from LE can be delivered into the vicinity of FA^36^. To examine if AnxA6 depletion in CHO M12 cells could influence the association of cholesterol with FA, the cellular distribution of the mCherry-tagged cholesterol biosensor D4H, which recognizes membrane domains enriched with cholesterol at the plasma membrane and organelles^37^, was analysed. Supporting its suitability to detect cholesterol in close vicinity of FA at cell edges, CHO-WT cells showed D4H staining in close proximity to EGFP-tagged vinculin or paxillin-containing FA structures at cell edges (Suppl. Figure 5), most likely reflecting D4H-positive peripheral recycling endosomes close to the plasma membrane^38^. Next, M12 and M12-A6ko cells were transfected with mCherry-D4H, starved overnight, serum-stimulated and stained with anti-pY861FAK (Fig. 8a). D4H staining confirmed cholesterol accumulation in perinuclear LE/Lys vesicles of NPC1 mutant cells. In these cells, many pY861FAK-positive structures throughout the cell body lacked overlap with strong punctate D4H staining (< 0.5% colocalization of pY861FAK with D4H; n = 30 cells), indicating low/limiting amounts of cholesterol in FA of NPC1 mutant cells that are not detectable using the D4H biosensor (see intensity profiles A-B, C-D in Fig. 8b, top panel). In contrast, in many M12-A6ko cells the colocalization of D4H with pY861FAK at cellular edges increased to ~ 12% (n = 10 cells, ***p < 0.001; see also intensity profiles for overlap of D4H and pY861FAK in E–F, G-H in Fig. 8b, bottom panel), implying increased amounts of cholesterol in or in close proximity of FA structures at cell edges in those cells. These findings suggest that the rescue of LE-Chol export in NPC1-deficient cells upon AnxA6 depletion^28,29^, possibly together with an improved functioning of exocytic pathways emanating from the Golgi and recycling endosomes^15,23–25^, and delivering cholesterol to pY861FAK-containing structures at or near the cell edges, even in the absence of LDL, supports cell migration.

## Discussion

Here we demonstrate that LDL stimulates migration and invasion of A431 squamous epithelial carcinoma and CHO fibroblasts, which is compromised upon genetic and pharmacological NPC1 inhibition or elevation of AnxA6 levels. Yet, NPC1 mutant cells can still establish multifactorial FA structures that contain activated FAK, vinculin and paxillin. LDL increases FA numbers at cell edges in controls, but not NPC1 mutant cells. Strikingly, AnxA6 depletion in NPC1 mutant cells increased their migratory behaviour and association of cholesterol with FA at cell edges, indicating that AnxA6 controls NPC1-independent LE-Chol transport routes that contribute to deliver cholesterol to FA to support cell migration.

Several animal studies substantiate LDL to promote cancer cell motility. In the hypercholesterolemic ApoE KO-mice and other mouse models, a high-fat/high cholesterol diet enhanced tumour growth and metastasis^39,40^. In contrast, proprotein convertase subtilisin/kexin type 9 deficiency, which lowers blood cholesterol due to an increased hepatic clearance of LDL, reduced melanoma metastasis in the liver^40^. This reduced metastatic potential was lost upon feeding a high cholesterol diet, indicating that nutrient-induced changes can override genetic mutations that lower the risk for cancer cell spreading. Indeed, hyperlipidemia delivers tumorigenic lipids, especially cholesterol, for tumour progression^41^ and in prostate and breast cancer, elevated LDL-cholesterol levels are associated with increased risk and progression^39,42^.

Cancer cells often cover the elevated demand for cholesterol via increased LDL endocytosis. On the other hand, LDL receptor downregulation impeded the growth of skin tumours^43^ and as shown here, LDL failed to stimulate migration in LDL receptor-deficient cells. Elevated LDL receptor levels in glioblastoma, leukemia and lung cancer may also explain the correlation between reduced plasma LDL-cholesterol and the high risk for various haematological and solid tumours^44^. In breast cancer cells, upregulated LDL receptor levels coincided with LDL stimulating cell migration in an ACAT-dependent manner^13^. Increased supply with LDL-derived cholesterol and cholesteryl ester accumulation supported pancreatic ductal adenocarcinoma and metastatic prostate cancer progression^11,14,45^.

Hence, LDL-derived cholesterol must exit LE/Lys to promote cell motility. In line with this study, depletion of lysosomal acid lipase or NPC1 inhibited LDL-inducible A431 cell migration^36^, indicating that cholesteryl ester hydrolysis, followed by LE-Chol export, enables LDL to stimulate migratory cell behaviour. Previous work^3,16,24,25^ and data presented here, using genetic and pharmacological NPC1 inhibition or AnxA6 overexpression, demonstrated that blocking LE-Chol export interfered with the migratory, but also invasive behaviour of several cancer cell lines. We here extend these observations to nutritional changes in local microenvironments, as increased delivery of LDL to LE/Lys stimulated A431 and CHO cell migration and invasion. Furthermore, when mimicking the three-dimensional complexity of the stromal tumour microenvironment, LDL-inducible A431 cell invasion was strongly reduced upon AnxA6 overexpression. Hence, besides NPC1, AnxA6 is another critical player in LE/Lys controlling the distribution of endocytosed and LDL-derived cholesterol to other cellular organelles, such as lipid droplets, and possibly the cell surface (see below), that are associated with invasive cancer cell behaviour.

LDL loading of A431 cells also increased EGF-inducible cell migration, supporting previous reports of a cholesterol-rich environment potentiating EGFR signalling^43,44^, and elevated LDL receptor levels stimulating integrin recycling^5^. This cooperativity was not observed in A431-A6 cells and we speculate that the scaffolding/targeting function of AnxA6 for the GTPase activating protein p120GAP and protein kinase Cα, both negative regulators of the EGFR/Ras/MAPK pathway, reduces EGF-driven signalling that promotes migration/invasion^20,31,33^, while AnxA6-mediated inhibition of LDL-derived LE-Chol egress^28,29^ interferes with LDL promoting cell motility. This multifunctionality of AnxA6 may also explain the improved efficacy of EGFR tyrosine kinase inhibitors to inhibit migration and invasion in AnxA6 expressing A431 cells^33^. Yet, AnxA6-dependent cooperativity of growth factors and lipoproteins may only occur in certain cells, as tyrosine kinase inhibitor treatment of triple-negative breast cancer cells caused AnxA6 upregulation and was associated with LE-Chol accumulation, possibly contributing to drug resistance^46^.

The ability of AnxA6 to inhibit LDL-inducible cell motility could be explained by AnxA6 associating with cholesterol-rich LE and recruiting the Rab7-GAP TBC1D15 to downregulate Rab7-GTP levels and block NPC1, but also StARD3-dependent LE-Chol export^28,29^. This pool of LDL-derived cholesterol accumulating in LE/Lys would then not be available to stabilize FA or support the motility of vesicles delivering FA components, such as integrins, to the cell surface.

In this context, Rab7 activity in the LE compartment may contribute to cancer cell motility^47,48^. Rab7-GTP levels are linked to nutrient availability^47^, making enhanced LDL endocytosis a potential driver to upregulate Rab7 activity, allowing augmented LDL-cholesterol export across LE/Lys membranes to support cell movement. This scenario would be strongly influenced by AnxA6 expression levels and its ability to control Rab7-GTP levels via the recruitment of the Rab7-GAP TBC1D15. Along these lines, TBC1D15 has been linked to oncogenic events^49^. Elevated StARD3 levels also correlate with metastasis, tumour recurrence and shorter survival in breast cancer and possibly colorectal, prostate, gastric and pancreatic cancer progression^50,51^.

The molecular machinery that connects LDL-derived cholesterol with FA dynamics remains to be fully understood. The oxysterol-binding protein-related protein 2 (ORP2/OSBPL2) has recently been identified to contribute to transfer LDL-cholesterol from NPC1-containing LE to FAK- and integrin-positive recycling endosomes close to the cell surface^38^. NPC1 depletion delayed delivery of LDL-containing vesicles from LE/Lys into the proximity of FA via a Rab8a-myosin5b-actin-dependent transport route, compromising FA dynamics at the leading edge of migrating cells^36^. Other points to consider include NPC1 inhibition to impact on Ca^2+^ homeostasis, other lipids besides cholesterol^52^ (Grewal, Rentero and Enrich, unpublished), elevate mTORC1 signaling and SREBP2 expression^53,54^, the latter being restored by AnxA6 deficiency in NPC1 mutants^28^. Nevertheless, despite low cholesterol levels at the plasma membrane, NPC1 mutant cells can establish multiprotein FA complexes and FAK signalling events appear operational. Yet, elevated and altered FA distribution may reflect the slower kinetics of integrin recycling^16^ and FA (dis-) assembly^36^ in these cells. However, upon LDL loading, only control cells displayed elevated FA numbers on cell edges, which might reflect the cellular response to enable LDL-inducible cell motility.

Interestingly, AnxA6 depletion in NPC1 mutant cells increased the association of the cholesterol biosensor D4H with FA at cell edges, indicating that AnxA6 controls NPC1-independent transport routes delivering cholesterol from LE to FA at the cell surface. This could occur via vesicular pathways emanating from LE/Lys, possibly via the recycling compartment, and involving Rab8 and ORP2^36,38^, or via the transfer of cholesterol via membrane contact sites between LE/Lys, the ER and the plasma membrane^28,29^. Alternatively, as the NPC1-mutant phenotype is associated with reduced cholesterol levels in the Golgi and recycling endosomes, AnxA6 deficiency could indirectly restore the functioning of de-regulated exocytic pathways and improve cholesterol transport from these sites to the cell surface^15,16,23–25^. This re-establishment of a cholesterol-enriched FA microenvironment in NPC1 mutant cells upon AnxA6 depletion may be the underlying cause for improved migration in these cells.

In addition, dysfunction of SNARE proteins SNAP23, Stx4 and Stx6 upon loss of NPC1 or AnxA6 overexpression^9,15^ imply LDL-derived cholesterol to participate and guide SNARE-dependent integrin trafficking for FA dynamics. Other events connected to LE-Chol export include the SNARE-dependent cell surface delivery of metalloprotease MT1-MMP and Src kinase from LE^55,56^. In fact, NPC1-mutant like phenotypes generated by the depletion of Endosomal Sorting Complexes Required for Transport (ESCRT) or Rab7, the latter also being inhibited by high AnxA6 levels^28^, interfered with Src translocation from LE to FA^57–59^, which is critical for FA turnover. Although we observed pY861FAK phosphorylation in FA of NPC1 mutant cells, NPC1 inhibition has been associated with highly upregulated Src protein levels^58,60^, indicating LE-Chol to fine-tune the functioning of Src kinase.

Other AnxA6-related aspects to be considered include the Ca^2+^-regulated and AnxA6-mediated inhibition of EGFR and Ras signalling at the plasma membrane^20,30,31,33^. Also, earlier reports linked extracellular AnxA6 with adhesive properties of metastatic cells^61,62^ and exosomal AnxA6 influenced BT-549 breast cancer cell motility, FA (dis-) assembly and FAK signalling^63^. In gastric and pancreatic cancers, AnxA6-containing extracellular vesicles from cancer-associated fibroblasts contributed to aggressiveness and metastasis, involving crosstalk with cell surface receptors or FAK signalling and cell surface presentation of integrins, respectively^64,65^. Finally, the previously reported association of AnxA6 with the Src family kinase Fyn and the focal adhesion kinase Pyk2 may also contribute to FA functioning^66^. We speculate that the ability of AnxA6 in LE to control the cellular distribution of LDL-derived cholesterol will likely govern the amount of AnxA6 proteins that function as scaffolds for signalling proteins in cholesterol-containing domains at the plasma membrane or that become secreted via exosomal pathways. Hence, understanding how changes in dietary LDL-cholesterol and cellular metabolic adaptions affect AnxA6 expression levels will provide further insights how critical players in the LE compartment that regulate cholesterol homeostasis, including NPC1, Rab7 and StARD3, contribute to cancer metabolism.

## Methods

### Reagents and antibodies

DMEM, Ham’s F-12, trypsin, L-glutamine, penicillin, streptomycin were from Invitrogen. Geneticin (G418), Mowiol and human recombinant EGF were from Merck. Bovine serum albumin (BSA), 4′,6-diamidino-2-phenylindole (DAPI), filipin, 2-mercaptoethanol, paraformaldehyde (PFA), puromycin, saponin, and U18666A were from Sigma. Rabbit polyclonal anti-NPC1 was from Abcam. Antibodies against total (mouse) and activated (rabbit) FAK (FAK, pY397FAK, pY861FAK) were from Invitrogen and BD Transduction Laboratories, respectively. Rabbit anti-β-actin was from Cell Signaling. Alexa Fluor-488 and -594 conjugated secondary antibodies were from Life Technologies. HRP-labelled secondary antibodies and SDS-PAGE molecular weight markers were from Cell Signaling.

LDL was isolated from donated, pooled blood samples from normal healthy donors (obtained from Red Cross, Melbourne, Australia; density 1.019–1.055 g/ml) by three sequential density gradient ultracentrifugations in KBr gradients^67^. All experimental protocols for the use of blood products purchased from the Red Cross (Material Supply Agreement no: 19-07NSW-1) for the isolation of plasma lipoproteins were approved by the local ethics committee of the University of New South Wales (HC190432) in accordance with the National Health and Medical Research Council’s (NHMRC) National Statement on Ethical Conduct in Human Research (2007).

Lipoprotein-deficient fetal calf serum (LPDS) was prepared by preparative ultracentrifugation^28^. Before experiments, LDL and LPDS were dialyzed extensively against PBS and stored at 4 °C until use. LDL protein concentration was determined^28^. EGFP-tagged expression vectors encoding wildtype and mutant NPC1 (P692S), paxillin, vinculin and mCherry-tagged cholesterol biosensor D4H were from Matthew P. Scott (Stanford University, California, USA), J. Victor Small (IMBA, Vienna, Austria), Guido Serini (University of Torino, Italy), Masashi Maekawa and Gregory D. Fairn (University of Toronto, Canada), respectively.

### Cell culture

Parental A431-WT and CHO-WT cell lines were obtained from the European Collection of Authenticated Cell Cultures (ECACC: #85090402 and #85051005). CHO ldlA, CHO M12 and CHO 2–2 were kindly provided by Monty Krieger (MIT, USA), Laura Liscum (Tufts University School of Medicine, USA) and Daniel S. Ory (Washington University, USA), respectively. The A431-WT cell line served to generate A431 cells stably expressing AnxA6 (A431-A6)^30,31^ and the CRISPR/Cas9-edited CHO M12 cell line lacking AnxA6 (CHO M12-A6ko) has been described^28^.

A431-WT, A431-A6 were grown in DMEM, and CHO-WT, CHO ldlA, CHO M12, CHO 2–2 and CHO M12-A6ko cells were grown in Ham’s F-12, together with 10% fetal calf serum (FCS), L-glutamine (2 mM), penicillin (100 U/ml) and streptomycin (100 µg/ml) at 37 °C, 5% CO_2_. A431-WT stably expressing scrambled or NPC1-targeting shRNA were grown in media containing 1.5 µg/ml puromycin. For transient transfections with fluorescently (EGFP, mCherry)-tagged vinculin, paxillin or D4H peptide, cells at 50% confluence were incubated with 1.5 µg DNA/ml using lipofectamine 2000 following manufacturer’s instructions.

### Suppression of NPC1 expression

1–2 × 10^6^ A431 cells were transfected with different combinations (total 1.5 µg) of four SureSilencing shRNA plasmids (SABiosciences) targeting human NPC1 (NM_000271.5) at position 1483–1503 (5’-GCACCAGGTTCTTGACTTACA-3’), 3061–3081 (5’-CTGCAATGCTTCAGTGGTTGA-3’), 3030–3050 (5’-GCTGTCGAGTGGACAATATCA-3’) and 3869–3889 (5’-GGAGCCACTCACGGATTAATA-3’), together with Lipofectamine 2000 as described^31^. After 48 h, cells were selected with 1.5 µg/ml puromycin. After 2 weeks, puromycin-resistant and NPC1-depleted colonies were identified by western blotting and filipin staining (Fig. 1d). The A431-NPC1-KD cell line (shRNA1-4) selected for migration assays was characterized by ≥ 35% NPC1 protein depletion. Stable A431 cells expressing scrambled shRNA (5’-GGAATCTCATTCGATGCATAC-3’) served as negative controls.

### Fluorescence microscopy

Cells were grown on coverslips for 48 h after transfection, and fixed with 4% PFA for 20 min at room temperature (RT), washed with PBS, permeabilized with 0.1% saponin for 10 min and blocked with 1% BSA for 5 min^28^. Coverslips were incubated with primary antibody diluted in 0.02% saponin, 0.1% BSA in PBS for 1 h at RT, washed intensively and then incubated with the adequate Alexa-488 and Alexa-594 conjugated secondary antibodies for 45 min at RT. After staining, coverslips were washed with PBS or DAPI-PBS solution, and mounted in Mowiol. Samples were visualized using a Leica TCS SP5 laser scanning confocal microscope equipped with a DMI6000 inverted microscope, blue diode (405 nm), Argon (458/476/488/496/514 nm), diode pumped solid state (561 nm), HeNe (594/633 nm) lasers and APO 63 × oil immersion objective lens or a Leica DMI6000B epifluorescence inverted microscope equipped with an HCX PLA APO 63 × oil immersion objective lens. Image analysis was performed with ImageJ (v1.47).

For filipin staining, cells grown on coverslips were fixed and permeabilized as above, incubated with 0.05 mg/ml filipin for 60 min, washed, dried and mounted in Mowiol^28^. Confocal microscopy was carried out using a Leica Spe-II confocal microscope equipped with APO 63 × oil immersion objective. Images were collected using Leica LAS AF software and image analysis and quantification of filipin staining intensity was performed with ImageJ (v1.47).

For the quantification of FA number, distribution and size, background subtraction from selected images (n ≥ 10–15 per condition and cell line) was performed as described^68^. Colocalization was determined using Pearson’s correlation coefficients^16^.

### Western blot analysis

Cell lysates were prepared, separated by SDS-PAGE and transferred to Immobilon-P (Millipore) as described^33^. Proteins were detected using their specific primary antibodies, followed by HRP-conjugated secondary antibodies and enhanced chemiluminescence detection (ECL, Perkin-Elmer). ImageJ was used for signal quantification^33^.

### Wound healing assays

5 × 10^5^ cells/6-well were seeded in triplicate and grown for 48–72 h until ∼90% confluence in media with 10% FCS or 10% LPDS. Cells were then treated ± U18666A (4 μg/ml), LDL (50 μg/ml), or both overnight. Scratches were made using a 200 μl pipette tip as described^16,24^. Cell debris was removed, treatment media was replenished and images were acquired after scratching (*t* = 0) and post-scratch every 4 h until cells completely covered the wound. Images were collected at 10 × magnification (Nikon Eclipse TS100 inverted microscope) with Leica Microsystems Digital Imaging. Image analysis was performed with ImageJ. The relative wound density (RWD %) and speed of wound closure (%/h) was calculated.

For scratch assays using the IncuCyte^33^, 1 × 10^4^ cells/well were seeded into 96-well plates, grown and treated ± U18666A, LDL, or both overnight as above. Then a single scratch was made in each well using the 96-pinblock Woundmaker™ (Essen Bioscience)^33^. Cell debris was removed, and treatment media was replenished. In some experiments, cells were starved in LPDS-containing media for 2 days, and LDL (50 μg/ml), EGF (10 ng/ml) or both was added after the scratch. Images were acquired using a 10 × objective on the IncuCyte ZOOM® (EssenBioscience) every 2–4 h. RWD (%) and cell front velocity (μm/h) was calculated^16,33^.

For pulse-chase experiments using the IncuCyte, cells were grown in media with 10% LPDS for 2 days, treated ± LDL (50 μg/ml) for 2 h before a single scratch was made. The media and cell debris were removed and replenished by LPDS-containing media. Images were acquired at 60 min intervals and RWD (%) was calculated.

### Matrigel invasion assays

A431-WT cell invasion was analyzed using BD BioCoat matrigel invasion chambers with 8 μm pore size polyethylene terephthalate membrane inserts (BD Biosciences)^24^. 6 × 10^4^ cells per insert were seeded on the upper chamber in serum-free medium and incubated ± U18666A (4 μg/ml). The lower chamber contained media with 10% FCS as chemoattractant. After 72 h, cells that had invaded the lower chamber were fixed and stained with Diff-Quik stain (Lab Aids). Cell migration with uncoated inserts served as control. Invasion and migration (± matrigel) was counted using ImageJ (v1.47). Images were captured using a Nikon Eclipse TS100 microscope and Leica Microsystems Digital Imaging such that each insert membrane contained six representative images of the entire membrane^24^.

### Organotypic invasion assay

7.5 × 10^4^/ml primary human fibroblasts were embedded in three-dimensional matrices of rat tail collagen type I. Rat tail tendon collagen solution was prepared by the extraction of tendons with 0.5 M acetic acid (≈2 mg/ml). The preparation of collagen type I from rat tails was approved and conducted in accordance with the Garvan/St Vincent’s Animal Ethics Committee guidelines (13/17, 14/06, 16/13, 19/10, and 19/13) and ARRIVE guidelines and in compliance with the Australian code of practice for care and use of animals for scientific purposes.

Detached, polymerized matrix (2.5 ml) in 35-mm dishes was allowed to contract for 6 days in DMEM, 10% FCS, until fibroblasts had contracted the matrix to ≈1.5-cm diameter^16,24,25^. Then 4 × 10^4^ A431-WT and A431-A6 cells were plated on top of the matrix and allowed to grow to confluence for 5 days. The matrix was then mounted on a metal grid and raised to the air/liquid interface, resulting in the matrix being fed from below with 10% LPDS-containing media ± LDL (50 μg/ml) that was changed every 2 days. After 14 days, the cultures were fixed using 4% PFA and processed for H&E staining. Invasion was calculated as the percentage of cells that invaded beyond ≈30 μm as a percentage of total cells in the assay^21,30^.

### Cholesterol determination

5 × 10^5^ cells/6-well were grown in media supplemented with 10% LPDS for 48 h before addition of U18666A (4 μg/ml), LDL (50 μg/ml), or both for additional 24 h. The media was removed, cells were washed, lipids were extracted as described^69^ and cholesterol was measured using the Amplex™ Red Cholesterol Assay Kit (Molecular Probes)^16,23^.

### Statistics

Statistical analysis was carried out using Microsoft Excel and Graph Pad Prism 9.2. Data represent means of at least 3 independent experiments with triplicate samples in each experiment, and unless stated otherwise, error bars show the standard deviation (SD). Statistical analysis was assessed using Mann–Whitney two-tailed, one- or two-way ANOVA with Tukey’s post-hoc tests as required. * = *p* < 0.05, ** = *p* < 0.01, *** = *p* < 0.001.

## Supplementary Information

Supplementary Information 1.

Supplementary Information 2.

## Abbreviations

ACAT: Acyl-CoA cholesterol acyltransferase
Anx: Annexin
Ca^2^^+^: Calcium
CHO: Chinese hamster ovary
EGF: Epidermal growth factor
EGFR: Epidermal growth factor receptor
ER: Endoplasmic reticulum
FA: Focal adhesion
FAK: Focal adhesion kinase
LDL: Low density lipoprotein
LE: Late endosomes
LPDS: Lipoprotein-deficient fetal calf serum
Lys: Lysosomes
NPC1: Niemann pick type C1
RWD: Relative wound density
SNARE: Soluble NSF Attachment Protein (SNAP) Receptor
SNAP23: Soluble N-ethylmaleimide-sensitive fusion protein 23
Stx: Syntaxin
wt: Wildtype
