# Supplementary material for: Annexin A6 and NPC1 regulate LDL-inducible cell migration and distribution of focal adhesions
Source: Sci Rep. 2022 Jan 12;12:596. doi: 10.1038/s41598-021-04584-y (PMC8755831; doi:10.1038/s41598-021-04584-y)

**Jose et al., 2021; Annexin A6 and NPC1 regulate LDL-inducible cell migration and distribution of focal adhesions**

**Figure S1A: Original exposures**

Cell lysates from CHO wildtype (WT; lane 1), CHO NPC1 mutant (M12; lane 2), and A431-WT stably expressing different combinations of four shRNAs targeting NPC1 (1-4, 1-3, 3-4, 1-2, lanes 3-6) or scrambled (scr, lane 7) shRNA were analyzed for NPC1 and ß-actin by western blotting as indicated. The top part of a single blot (>50 kD) was analyzed for NPC1, and the bottom part (<50 kD) for ß-actin.


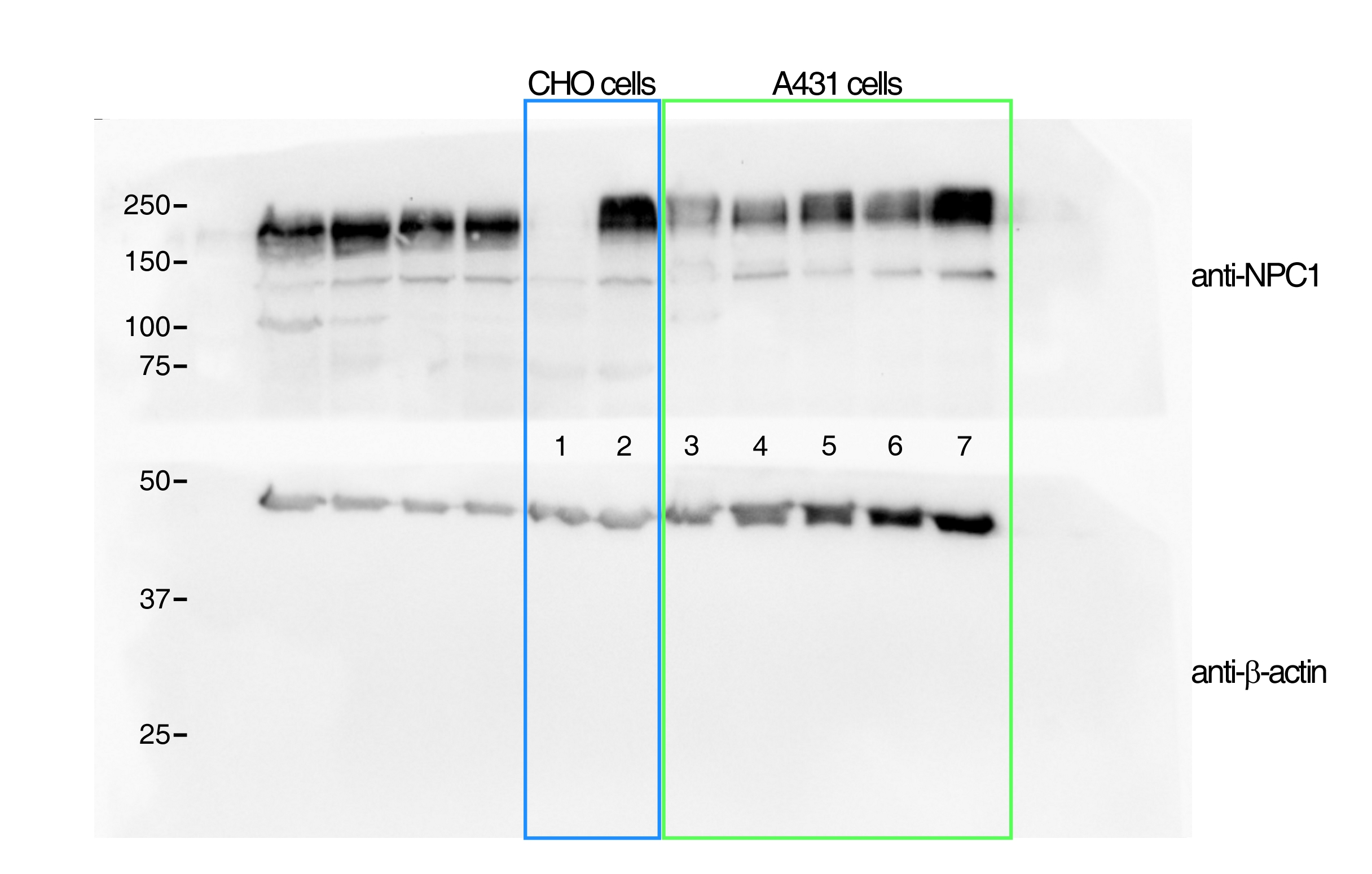

Supplement: Supplementary file 1 — Supplementary Information 1. [file 41598_2021_4584_MOESM1_ESM.docx]
