# Supplementary material for: Annexin A6 and NPC1 regulate LDL-inducible cell migration and distribution of focal adhesions
Source: Sci Rep. 2022 Jan 12;12:596. doi: 10.1038/s41598-021-04584-y (PMC8755831; doi:10.1038/s41598-021-04584-y)
Supplement: Supplementary file 2 — Supplementary Information 2. [file 41598_2021_4584_MOESM2_ESM.pdf]

Annexin A6 and NPC1 regulate LDL-inducible cell migration and distribution of focal adhesions

Jaimy Jose, Monira Hoque, Johanna Engel, Syed S. Beevi, Mohamed Wahba, Mariya Georgieva, Kendelle Murphy, William E. Hughes, Blake J. Cochran, Albert Lu, Francesc Tebar, Andrew J. Hoy, Paul Timpson, Kerry-Anne Rye, Carlos Enrich, Carles Rentero, Thomas Grewal

Supplementary information

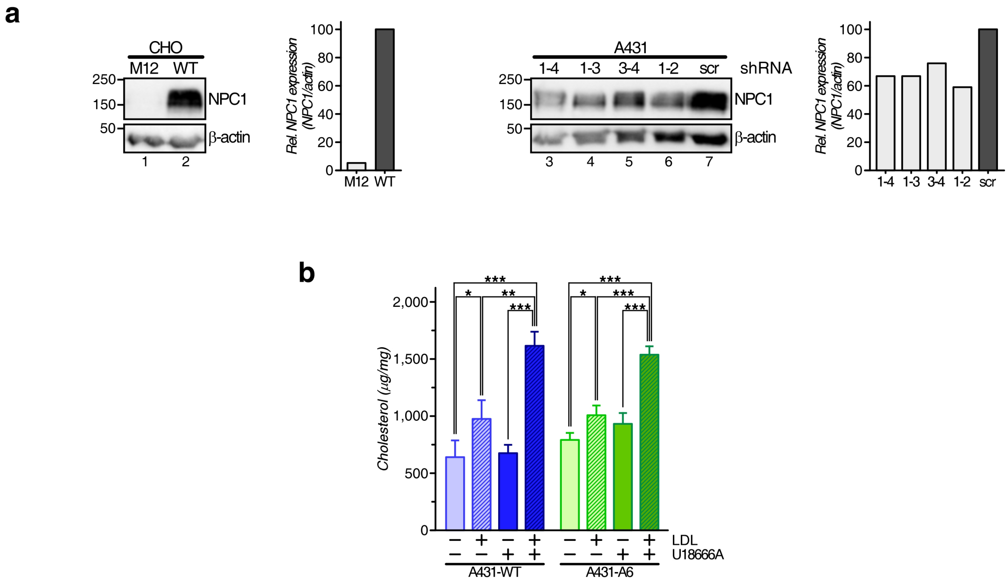

Supplementary Figure 1: (a) Cell lysates from CHO wildtype (WT), CHO NPC1 mutant (M12) (lane 1-2), and A431-WT stably expressing different combinations of four shRNAs targeting NPC1 (1-4, 1-3, 3-4, 1-2) (lane 3-6) or scrambled (scr) shRNA (lane 7) were analyzed for NPC1 and ß-actin by western blotting as indicated. Relative NPC1 expression (NPC1/ß-actin) was calculated. The A431-WT cell line expressing shRNAs 1-4 (lane 3) was used for further studies. **(b)** Cholesterol levels in A431-WT and A431-A6 cells incubated ± LDL (50 µg/ml) for 24 h in the presence or absence of the pharmacological NPC1 inhibitor U18666A. A431-WT and A431-A6 cells were seeded in 6-well plates and grown in media supplemented with 10% LPDS for 48 h before addition of LDL (50 µg/ml), U18666A (4 µg/ml), or both for additional 24 h. Cells were collected, lipids were extracted, and the amount of cholesterol (µg/mg) was determined and normalized to total cellular protein as described in Methods. The mean ± SEM from three independent experiments with triplicate samples is given. * $p < 0.05$, ** $p < 0.01$, *** $p < 0.001$; two-way ANOVA with Tukey's post-hoc test.

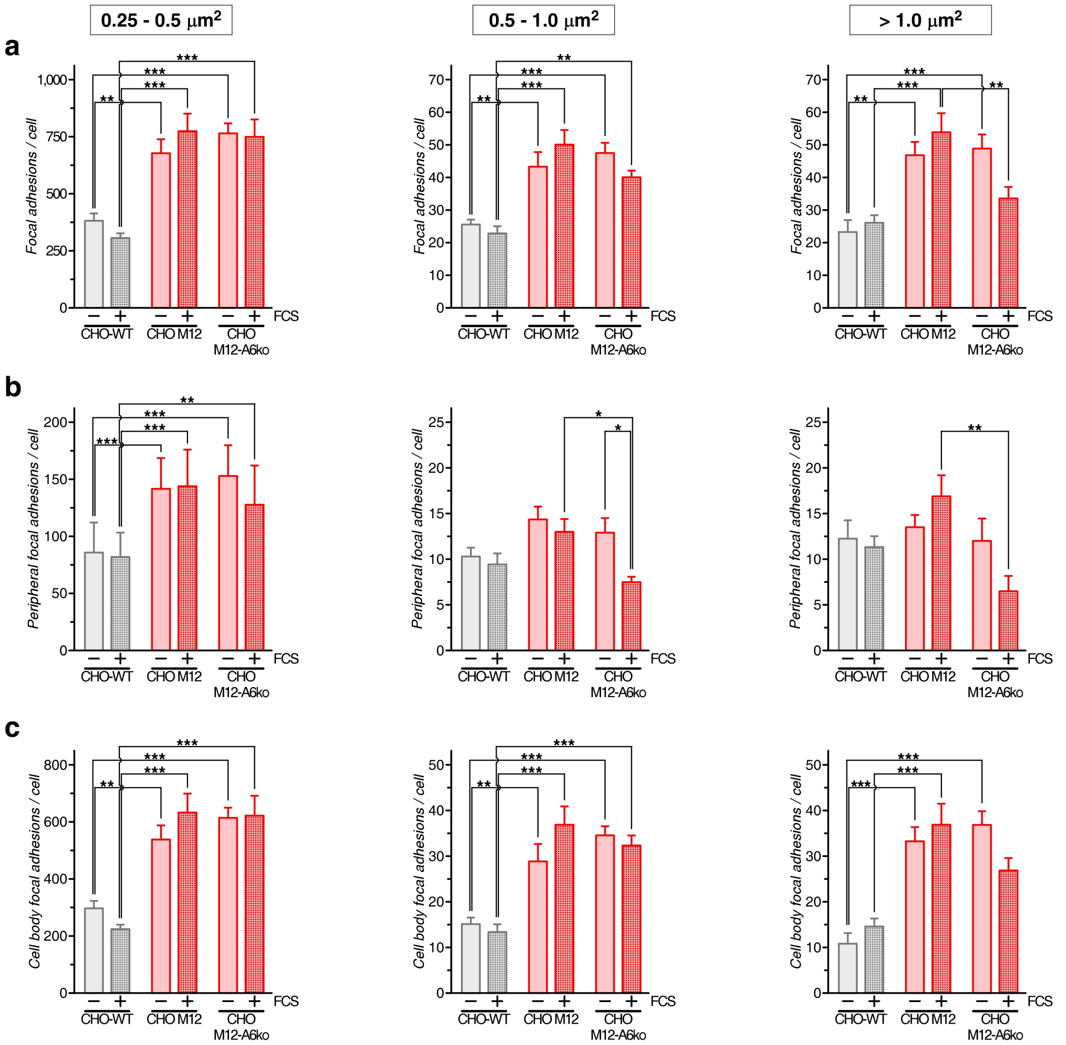

Supplementary Figure 2: Distribution and size of focal adhesions in CHO-WT, M12 and M12-A6ko cells. **(a-b)** CHO-WT, CHO M12 and CHO M12-A6ko cells were starved overnight,-serum-activated with 20% FCS for 45 min as indicated (-, +), fixed and stained for phosphorylated FAK (pY861FAK; see Fig. 5c-d). The number of small (0.25 - 0.5 μm^2), medium (0.5 – 1 μm^2) and large (> 1 μm^2) focal adhesions per cell (total), at the cell edge and throughout the cell body was quantified**.** 20-27 cells per condition and cell line were counted. The mean ± SEM is given. * $p < 0.05$, ** $p < 0.01$, *** $p < 0.001$, two-way ANOVA with Tukey's post-hoc test.

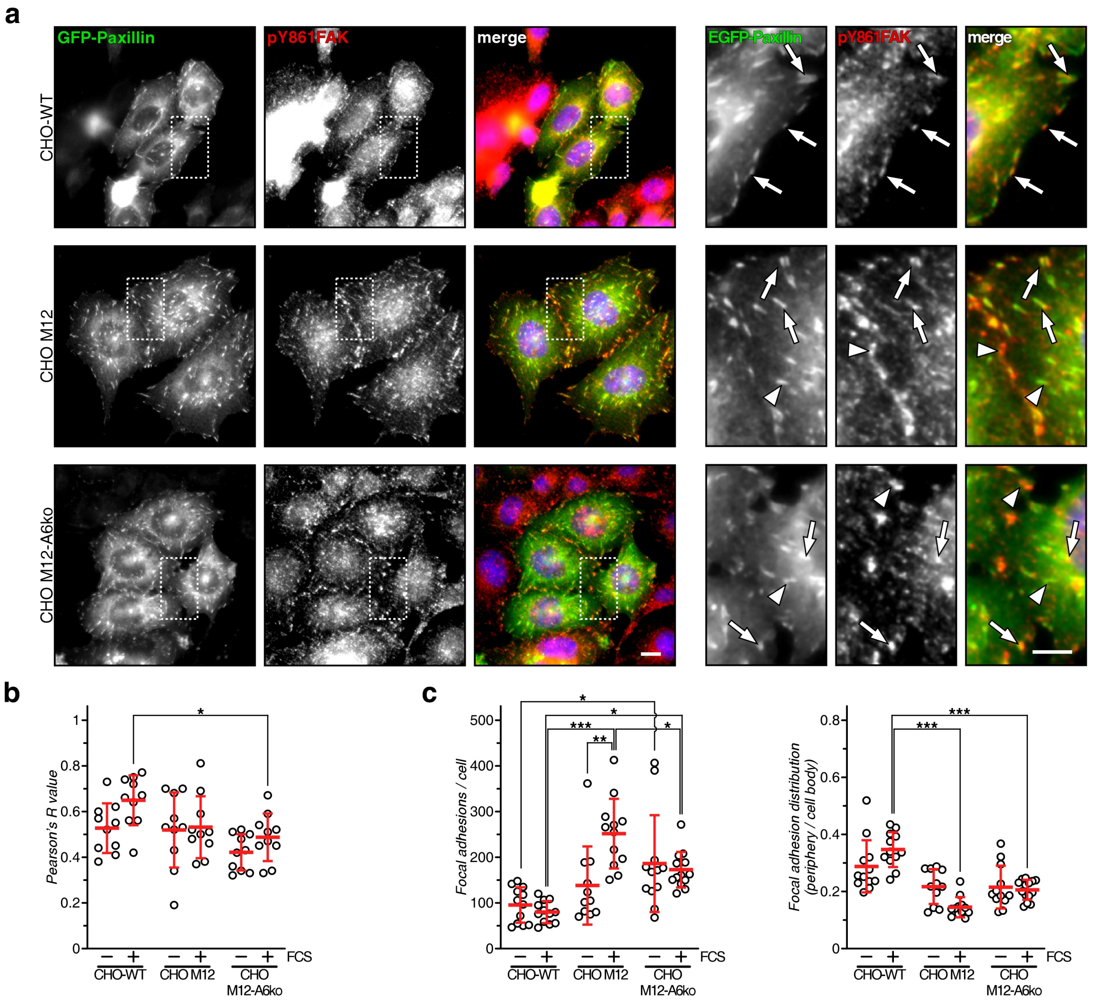

Supplementary Figure 3: Distribution of FA marker paxillin in CHO-WT, CHO M12 and CHO M12-A6ko cells. **(a)** CHO-WT, CHO M12 and CHO M12-A6ko cells ectopically expressing EGFP-paxillin were starved overnight, serum-stimulated (20% FCS) for 45 min, fixed and stained for phosphorylated FAK (pY861FAK, red) as indicated. The merged images and enlarged regions of interest are shown. Arrows indicate colocalization of EGFP-paxillin and pY861FAK. Arrowheads point at EGFP-paxillin or pY861FAK that do not colocalize. Bar is 10 µm and 2 µm for enlarged insets. **(b)** Colocalization of EGFR-paxillin and pY891FAK was quantified (Pearson's R value; 20-30 cells per condition and cell line were quantified. **(c)** Focal adhesions (>0.25 μm^2) per cell (left panel) and the ratio of focal adhesions at the cell edge vs. cell body (right panel) was quantified**.** 7-13 cells per condition and cell line were counted. The mean ± SD is given. * $p<0.05$, ** $p<0.01$, *** $p<0.001$; two-way ANOVA with Tukey's post-hoc test.

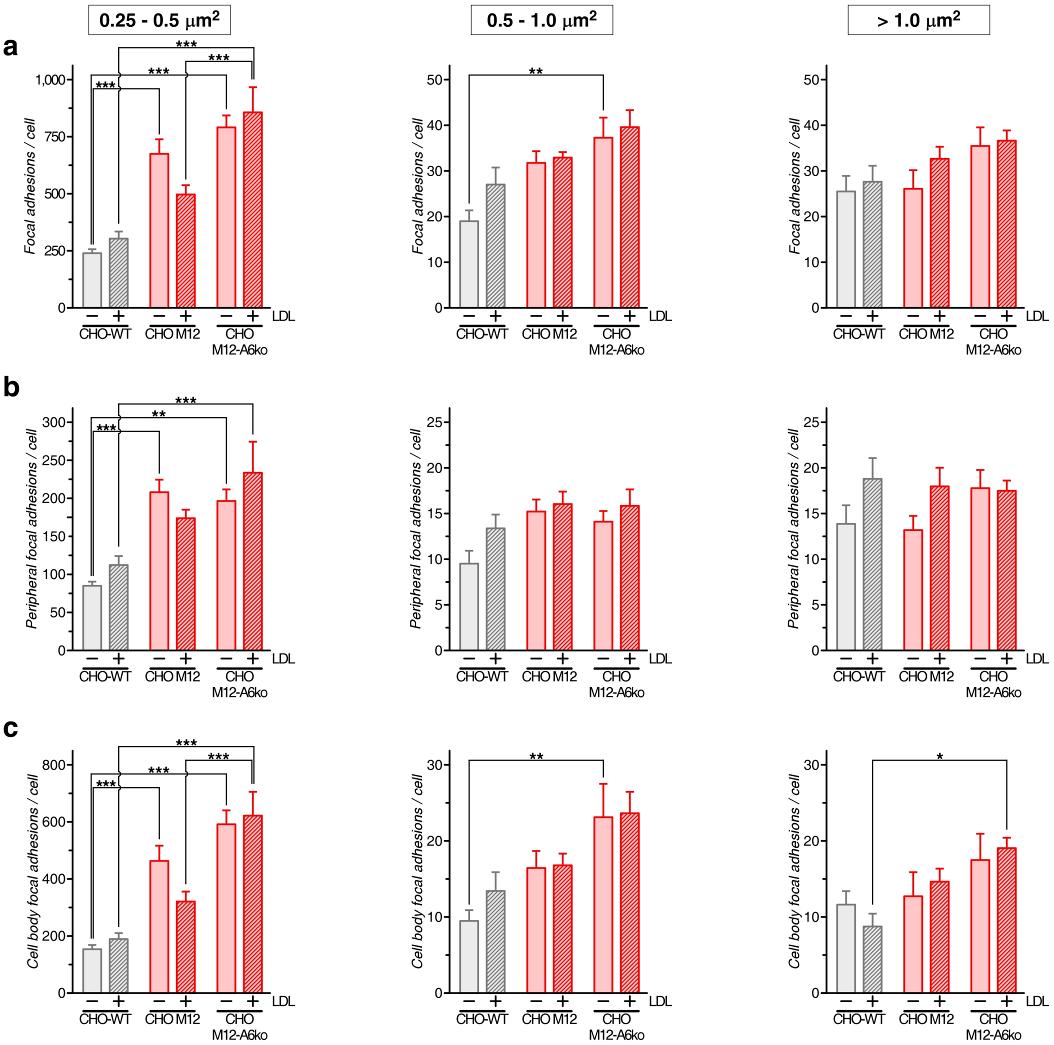

Supplementary Figure 4: Distribution and size of focal adhesions in LDL-loaded CHO-WT, M12 and M12-A6ko cells. **(a-b)** CHO-WT, CHO M12 and CHO M12-A6ko cells were grown in 10% LPDS-containing media for 2 days and then treated ± LDL (50 μg/ml) for 4 h. Cells were fixed and immunolabeled with anti-pY861FAK. The number of small (0.25 - 0.5 μm^2), medium (0.5 – 1 μm^2) and large (> 1 μm^2) focal adhesions per cell (total), at the cell edge and throughout the cell body was quantified in control and LDL-loaded cells as indicated (-, +). 16-21 cells per condition and cell line were counted. The mean ± SEM is given. * $p<0.05$, ** $p <0.01$, *** $p < 0.001$; two-way ANOVA with Tukey's post-hoc test.

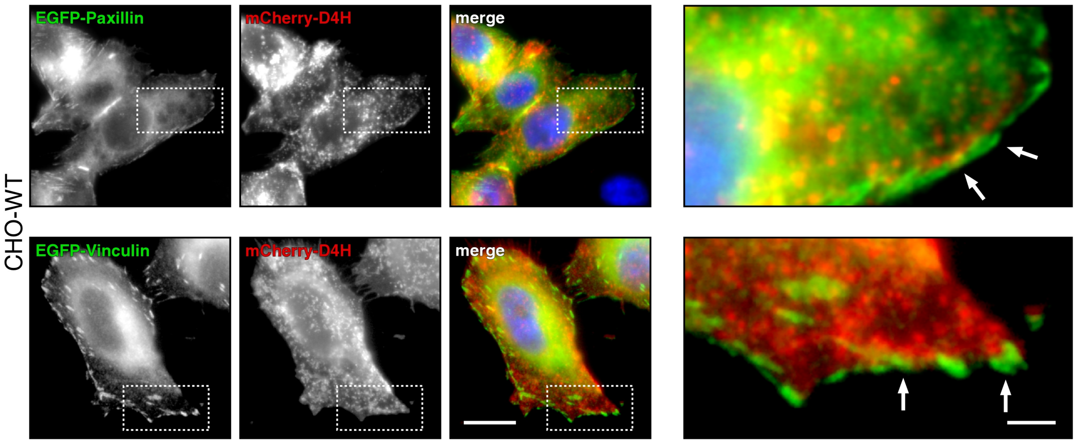

Supplementary Figure 5: Association of cholesterol with FA structures in CHO-WT cells. **(a-b)** CHO-WT cells ectopically expressing the cholesterol biosensor mCherry-D4H (red) together with EGFP-tagged paxillin (green, upper panel) or vinculin (green, lower panel) were starved overnight, serum-stimulated (20% FCS) for 45 min, fixed and analyzed by microscopy as described in Methods. The merged images are shown. Arrows point at paxillin- or vinculin-containing FA structures at cell edges with D4H in the vicinity. Bar is 10 μm and 2 μm for enlarged inset.
